# Clay Edges Are Dynamic Proton-Conducting Networks Modulated by Structure and pH

**DOI:** 10.1021/acs.jpclett.5c03748

**Published:** 2026-02-23

**Authors:** Yixuan Feng, Xavier R. Advincula, Hongwei Fang, Christoph Schran

**Affiliations:** † State Key Laboratory of Hydro-science and Engineering, Department of Hydraulic Engineering, 12442 Tsinghua University, Beijing 100084, China; ‡ Yusuf Hamied Department of Chemistry, 2152 University of Cambridge, Lensfield Road, Cambridge CB2 1EW, U.K.; § Cavendish Laboratory, Department of Physics, 2152 University of Cambridge, Cambridge CB3 0HE, U.K.; ∥ Lennard-Jones Centre, 2152 University of Cambridge, Trinity Ln, Cambridge CB2 1TN, U.K.

## Abstract

Montmorillonite, a ubiquitous clay mineral, plays a vital role in geochemical and environmental processes due to its chemically complex edge surfaces. However, the molecular-scale acid–base reactivity of these interfaces remains poorly understood due to the limitations of both experimental resolution and conventional simulations. Here, we employ machine learning potentials with first-principles accuracy to perform nanosecond-scale molecular dynamics simulations of montmorillonite nanoparticles across a range of pH. Our results reveal clear amphoteric behavior, with edge sites undergoing protonation in acidic environments and deprotonation in basic conditions, accompanied by pH-dependent surface charge regulation. Even at neutral pH, spontaneous and directional proton transfer events are common, proceeding via both direct and solvent-mediated pathways. These findings demonstrate that montmorillonite edges are not static arrays of hydroxyl groups but dynamic, proton-conducting networks whose reactivity and charge state are modulated by local structure and solution conditions. This work offers a molecular-level framework for understanding proton transport and buffering in clay–water systems, with broad implications for catalysis, ion exchange, and environmental remediation.

## Graphical abstract

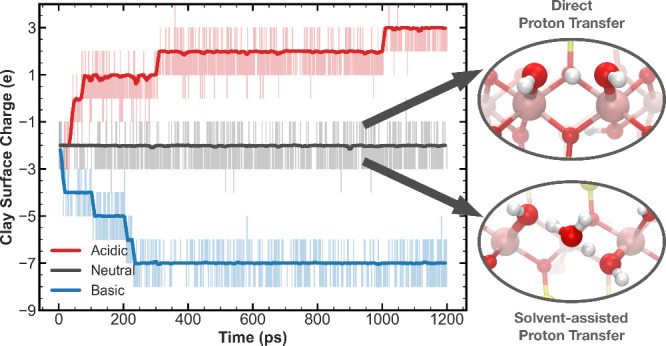

Clay minerals are ubiquitous in terrestrial and marine environments and play an essential role in a wide range of geochemical and environmental processes, including nutrient cycling, metal mobilization, ice nucleation, and sediment transformation.^1–5^ In technological applications, clay-based materials are widely employed in pollution control, water purification, and radioactive waste containment owing to their exceptional cation exchange capacity and adsorption performance.^6–8^ To fully elucidate the role of clay minerals in these processes and to optimize their functional applications, it is crucial to develop a detailed understanding of their adsorption capabilities,^9^ dissolution and growth kinetics,^10^ and aggregation behavior.^11^ Underlying all of these phenomena is the surface chemistry of clay minerals, particularly their acid–base properties and surface proton dynamics.

Among these minerals, montmorillonite—a swelling smectite with a layered aluminosilicate structure—has emerged as one of the most extensively studied clays due to its high specific surface area, thermal and mechanical stability, and intrinsic structural charge heterogeneity.^12,13^ These properties render montmorillonite a highly effective cation sorbent with broad applications in heavy metal removal and contaminant immobilization. However, despite decades of research, a fundamental molecular-level understanding of montmorillonite surface reactivity remains elusive. The basal surfaces, dominated by siloxane sheets, are chemically inert and largely pH-independent.^14^ In contrast, edge surfaces expose amphoteric hydroxyl groups that, although less abundant, are central to proton exchange, metal complexation, and mineral dissolution, particularly under variable pH conditions.^15–17^ The structural heterogeneity and chemical complexity of these edge sites, particularly in environmentally relevant aqueous systems, continue to present major challenges for direct characterization, thus hindering mechanistic understanding.

Extensive experimental efforts have been made to probe the acid–base reactivity of montmorillonite surfaces, particularly through acid–base titration.^18–20^ However, despite careful sample pretreatment and modeling efforts, it remains difficult to independently determine the density and intrinsic acidity constants of individual surface functional groups. Instead, surface protonation is often inferred from macroscopic titration curves using surface complexation models (SCMs), which rely on simplified assumptions and cannot unambiguously resolve the contributions of specific edge sites.^21–23^ The structural and chemical heterogeneity of montmorillonite edge surfaces, encompassing the diversity of Si- and Al-associated hydroxyl groups as well as the effects of isomorphic substitutions, further complicates experimental characterization and molecular modeling efforts.^24^

To overcome the limitations of experimental approaches in resolving surface protonation at the atomic level, *ab initio* molecular dynamics (AIMD) simulations have been widely employed to investigate the acid–base behavior of clay mineral edges.^25^ Multiple studies have calculated the intrinsic acidity constants (p*K*_a_) of individual hydroxyl groups on montmorillonite, kaolinite, and pyrophyllite edge surfaces, demonstrating how site-specific chemistry is modulated by structural factors such as coordination state and isomorphic substitution.^26–29^ These simulations consistently confirm the amphoteric nature of edge sites, and suggest that substitutions in both tetrahedral and octahedral sheets can significantly alter local acid strength, often elevating the p*K*_a_ and stabilizing the protonated state. Beyond p*K*_a_ estimation, AIMD studies have also explored spontaneous proton transfer (PT) dynamics and hydration structures at fully solvated clay edges.^30,31^ These works reveal that surface reactivity strongly depends on crystallographic orientation and interfacial hydrogen-bond networks. For example, the (010) edge of pyrophyllite has been shown to support spontaneous proton exchange between adjacent hydroxyl groups via water-mediated pathways, while the (110) surface remains largely inert under comparable conditions. Interlayer and micropore water can further stabilize charged species, emphasizing the need to consider the full solvation environment.

Despite these advances, current simulations remain limited by spatial and temporal scales. Most AIMD studies probe only isolated surface reactions over picosecond time scales and predominantly under neutral pH conditions, providing limited access to correlated proton dynamics, extended proton transfer pathways, or the evolution of surface charge states. As a result, direct molecular-level insight into how amphoteric surface chemistry, proton transfer, and surface charge regulation are coupled at fully hydrated clay nanoparticles, which represent the relevant reactive units in natural and engineered environments, remains largely absent. Addressing this gap requires modeling strategies that retain quantum-level accuracy for reactive edge groups while enabling nanosecond-scale sampling of realistic, solvated mineral surfaces across a range of pH.

Recent developments in machine learning potentials (MLPs) have provided a powerful and efficient framework for simulating chemically complex clay–water interface systems. By learning the potential energy surface from first-principles data, MLPs dramatically reduce computational cost while preserving *ab initio* accuracy, thereby enabling nanosecond-scale simulations of large, solvated mineral surfaces.^32^ This approach has already proven effective in modeling structural and mechanical properties of phyllosilicates such as kaolinite^33,34^ and pyrophyllite,^35^ yielding results consistent with both density functional theory (DFT) and experimental data. Beyond static structure, MLPs have been used to investigate dynamic interfacial phenomena such as water layering, hydrogen-bond dynamics, and ion exchange, achieving temporal and spatial resolution beyond the reach of conventional AIMD simulations.^36,37^ Furthermore, MLPs retain a fully reactive description at near quantum-level accuracy. Although not yet applied to proton transport in clays, recent studies have successfully captured acid–base reactions and proton transport at graphitic and oxide surfaces, as well as within confined aqueous environments,^38–41^ offering detailed insights into bond-breaking, charge delocalization, and solvation effects. These advances show that MLPs are uniquely suited to bridge the current knowledge gap in clay edge chemistry by capturing both the structural heterogeneity and the dynamic proton exchange processes at the mineral–water interface over extended time scales. In this context, MLP-based simulations offer a promising path forward to resolve the coupled mechanisms of surface acid–base reactivity and PT in realistic clay nanoparticle systems.

In this study, we leverage the accuracy and efficiency of MLPs to investigate the surface chemistry of montmorillonite in aqueous environments. Our simulations reveal the amphoteric nature of clay edge surfaces and show that proton exchange between edge functional groups and the aqueous phase gives rise to dynamic surface charge regulation across a range of pH. We further demonstrate that spontaneous proton transfer between neighboring surface groups is prevalent under neutral conditions, giving rise to dynamic proton-conducting networks whose activity is subsequently shown to be modulated by solution pH and isomorphic substitution. These results provide a molecular-level framework for understanding how acid–base reactivity, proton mobility, and surface charge buffering are coupled at clay–water interfaces.

Montmorillonite is a 2:1 dioctahedral phyllosilicate clay mineral characterized by isomorphic substitution of Al by Mg in its octahedral sheet, described by a general formula of M_*y*_^+^[Si_8_]­[Al_4–*y*_Mg_*y*_]­O_20_(OH)_4_. Given the dominant role of edge sites in governing acid–base reactivity of clays, as established in prior experimental and theoretical studies, our simulations focused on capturing the structure and chemical dynamics of montmorillonite edge surfaces in aqueous environments. Informed by atomic force microscopy (AFM) measurements and molecular dynamics simulations, the (110) and (010) edge surfaces—commonly referred to as the AC and B edges^42^—are identified as the most frequently exposed terminations, accounting for approximately 60% and 20% of the total edge area, respectively.^43,44^ To reflect this distribution, we constructed hexagonal-shaped montmorillonite nanoparticles comprising four AC and two B edges (Figure 1a). While ideal hexagonal symmetry is rarely observed in natural specimens, this construction enables simultaneous exploration of diverse edge types and their potential cooperative interactions within a single simulation setup. The atomic structures of the B and AC edge terminations are shown in Figure 1b,c, using minimal yet structurally representative models commonly employed in atomistic simulations of clay nanoparticles.^30,31,45–47^ All broken bonds at the edges were chemically terminated with hydroxyl or hydrogen groups to maintain local charge neutrality. To investigate the role of compositional disorder, three Mg-for-Al isomorphic substitutions were randomly introduced into each nanoparticle, and three distinct substitution patterns were considered (Mont.1, Mont.2, and Mont.3; see Supplementary Section S1). To examine the pH-dependent reactivity of edge sites, each nanoparticle was simulated in aqueous environments prepared at initial acidic (pH 0.44), neutral (pH 7), and basic (pH 13.56) conditions (Figure 1d–f), including respective counterions to retain charge neutrality. These pH values represent the equivalent bulk pH associated with the initial concentrations of hydronium or hydroxide ions in the simulation cell and are provided as a reference for comparison. During the simulations, no external buffering or ion exchange was imposed to maintain a fixed pH; instead, proton exchange between the aqueous phase and the clay edge surfaces was allowed to proceed freely, leading to time-dependent changes in the effective acidity of the solution. Accordingly, the terms acidic, neutral, and basic environments are used throughout to denote these composition-defined conditions rather than strictly controlled macroscopic pH values. To enable a detailed exploration of chemical reactivity in these systems, a MLP was developed using the MACE framework^48^ and trained on revPBE-D3 reference data (see Methods). The training data set encompassed bulk aqueous solutions and clay–water interface structures under a range of pH conditions, allowing the model to accurately reproduce energies and forces across diverse chemical environments. The resulting potential reliably captures interfacial energetics, clay lattice properties, the structure of aqueous phases, and key proton transfer thermodynamics (see Supplementary Sections S1 and S2). Nanosecond-scale molecular dynamics simulations using this MLP enabled us to examine the acid–base reactivity of montmorillonite edge surfaces in various aqueous environments, capturing their dynamic protonation states and associated PT behavior under chemically realistic conditions.

**Figure 1:**
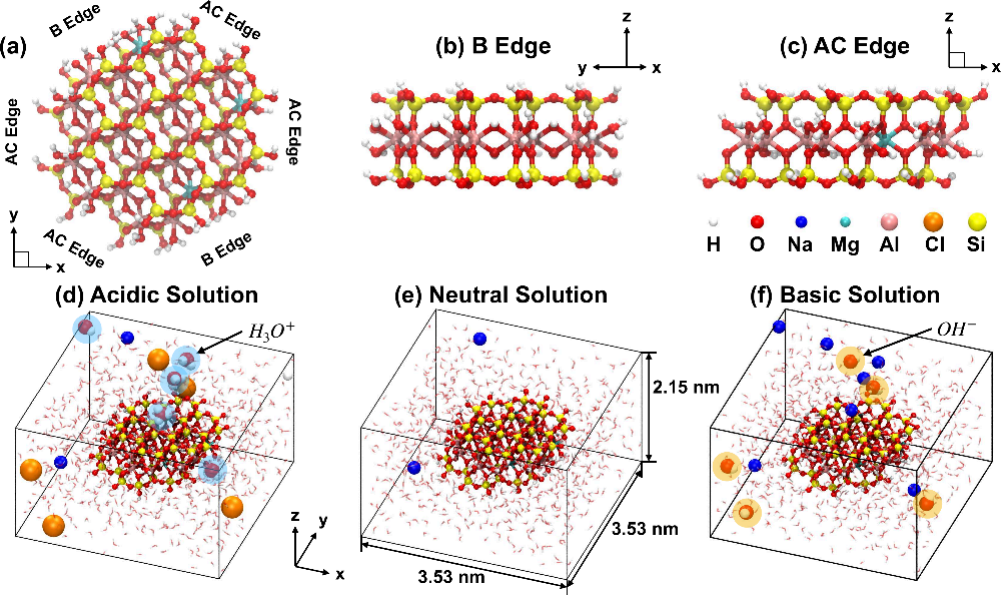
Snapshots of the simulation system comprising a montmorillonite nanoparticle surrounded by aqueous solution. (a) Top view of the clay nanoparticle. (b) Side view perpendicular to the B edge. (c) Side view perpendicular to the AC edge. (d–f) Representative snapshots of the nanoparticle in aqueous solution under different initial pH conditions: (d) acidic (pH 0.44), (e) neutral (pH 7), and (f) basic (pH 13.56). Coordinate axes are provided to indicate the viewing directions.

### Amphoteric and Dynamic Nature of Edge Surfaces

To explore the acid–base reactivity of montmorillonite edge surfaces, we first examined the dynamic protonation behavior of nanoparticles under acidic, neutral, and basic conditions. Figure 2 presents representative results for one of the studied montmorillonite structures; analogous behavior was observed for the other two nanoparticle configurations (see Supplementary Section S3). In Figure 2a, the solution-phase net proton excess, defined as the number of hydronium ions minus hydroxide ions, starts at about +5 under acidic conditions and about −5 under basic conditions. As the simulation proceeds, the mean absolute value in both cases gradually decreases and approaches zero, indicating progressive proton uptake by the nanoparticle in acidic environments and proton release in basic environments. The evolution of the aqueous proton population and the surface charge of the montmorillonite nanoparticle represent two complementary views of the same proton-exchange process, as shown in Figure 2a,b. Changes in the number of hydronium and hydroxide ions in solution are necessarily mirrored by protonation and deprotonation events at the edge surfaces, leading to a dynamic redistribution of charge between the aqueous phase and the nanoparticle. Under acidic conditions, proton uptake at edge functional groups increases the positive surface charge density, whereas under basic conditions, deprotonation of surface hydroxyls enhances the net negative surface charge. Together, these results demonstrate that the clay nanoparticle and the surrounding solution form a coupled reactive system in which reversible proton exchange gives rise to pH-dependent surface charge regulation and buffering behavior, reflecting the amphoteric character of the nanoparticle. This behavior is consistent with previous *ab initio* simulations, which inferred amphoteric character from a limited number of proton transfer events at specific edge sites under neutral conditions.^30^ In contrast, our simulations evaluate protonation behavior across a range of pH environments, allowing a more comprehensive and chemically direct assessment of the ampotheric nature of montmorrilonite based on the net acid–base response of the particle. This macroscopic amphoteric trend is also consistent with earlier titration and coagulation experiments on montmorillonite suspensions, which suggested pH-dependent charge development at edge sites.^15^ While this qualitative behavior is robust across structural variations, the kinetics of protonation and deprotonation differ significantly: the average time required to lose five protons in basic solution was approximately 179 ± 46 ps, whereas acquiring five protons in acidic solution occurred no earlier tha 634 ps and was not completed within 1.2 ns in some cases (see Supplementary Section S3).

**Figure 2:**
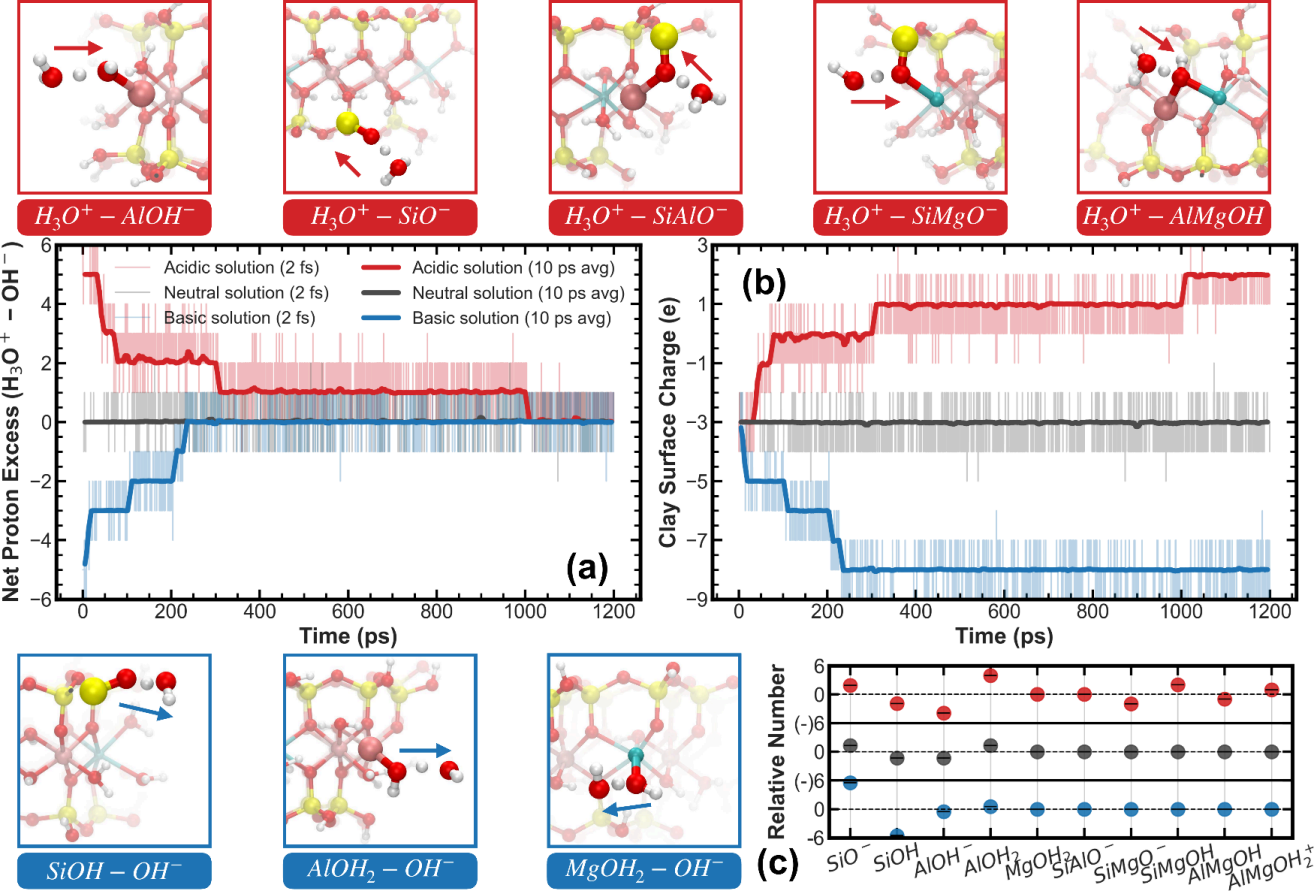
Protonation and deprotonation behavior of the montmorillonite nanoparticle in different aqueous environments. (a) Time evolution of the net proton excess in the aqueous phase (defined as the number of hydronium ions minus hydroxide ions) over 1200 ps. (b) Time evolution of the net surface charge of the montmorillonite nanoparticle, derived from the intrinsic negative structural charge of the clay due to isomorphic substitution, together with protonation and deprotonation of the nanoparticle through proton exchange with the aqueous solution. Transparent curves represent data sampled every 2 fs; solid lines denote a 10 ps average. (c) Relative abundance of various surface functional groups during the final 200 ps, compared to their initial populations. Error bars are included but smaller than the symbol size. In all panels, colors denote the solution pH: red for acidic, black for neutral, and blue for basic conditions. Top panel: Surface groups that gain protons from hydronium ions under acidic conditions. Bottom panel: Surface groups that donate protons to hydroxide ions under basic conditions. Arrows indicate the direction of proton transfer, with representative surface reactions labeled below each group pair. Results shown correspond to one representative montmorillonite nanoparticle; analogous behavior for the other two configurations is provided in the Supporting Information.

Trajectory analysis, integrating results from all nanoparticle configurations, revealed distinct sets of reactive surface groups under different conditions. In acidic environments, protonation occurred primarily at negatively charged or undercoordinated sites, including −AlOH^–^, −SiO^–^, −SiAlO^–^, −SiMgO^–^, and −AlMgOH. In basic environments, deprotonation involved groups such as −SiOH, −AlOH_2_, and −MgOH_2_ (Figure 2, top and bottom panels). These differences reflect not only the intrinsic acid–base properties of the functional groups but also their abundance and spatial distribution. Sites reactive toward hydroxide ions are more prevalent and broadly distributed across both AC and B edges. In contrast, groups readily protonated by hydronium ions are more localized, primarily occurring along B edges and AC edges that contain isomorphic substitutions. Among these, the −AlMgOH group is of particular interest. Although rarely considered in previous structural models, this site at the intersection of two AC edges frequently undergoes protonation in our simulations. However, its reactivity appears contingent upon the presence of Mg substitution at one of the adjacent Al sites; systems lacking this substitution show no clear protonation of the corresponding −AlAlOH group. These findings highlight the critical role of isomorphic substitution in modulating local acid–base behavior, later analyzed in more detail. The proton affinities of other active sites, including −AlOH^–^, −SiAlO^–^, and −SiMgO^–^, are well supported by prior computational studies.^44,49^ While these studies predicted high proton affinity based on free energy calculations or Fukui function analysis, our simulations directly capture the protonation of these sites by hydronium ions under acidic conditions, providing dynamic, atomistic confirmation of their Brønsted basicity.

Figure 2c presents representative results quantifying the relative population changes of key surface groups during the final 200 ps. In acidic conditions, protonated species such as −AlOH_2_, −SiMgOH, and −AlMgOH_2_^+^ became more abundant, forming not only via protonation by hydronium ions but also through PT from −SiOH. Conversely, in basic solutions, −SiOH groups transferred protons both to hydroxide ions in solution and to nearby −AlOH^–^ groups. These observations highlight that proton exchange occurs not only between surface sites and the bulk solution but also among surface groups themselves. In neutral solutions, the decrease in −SiOH and the increase in −AlOH_2_ suggest that spontaneous PT occurs between these two surface groups. While these events are consistent with previous simulation studies that reported proton transfer between −SiOH and −AlOH^–^ groups at clay edges,^30,31^ those works captured only isolated, short-lived PT events over limited simulation time scales. For instance, Churakov^31^ observed a reversible PT between these sites within several picoseconds, while Suter et al.^30^ reported unidirectional transfer without reversal under specific hydration environments. In contrast, our long-time scale simulations reveal a statistically favored net deprotonation of −SiOH and protonation of −AlOH^–^ groups under neutral conditions, suggesting that this directional PT process is not merely transient, but reflects a thermodynamically preferred configuration. This spontaneous PT is accompanied by persistent fluctuations in the net proton excess of the aqueous phase, indicating that water molecules, in the form of hydrated hydronium ions and hydroxide ions, play a role in these PT events. Although the probability of these events occurring is generally low, they are indicative of the dynamic nature of proton exchange on the surface (see Supplementary Figure S9).

Together, these results demonstrate that the amphoteric behavior of montmorillonite edge surfaces arises from dynamic, site-specific proton exchange involving both the aqueous phase and neighboring surface groups. Rather than remaining in fixed protonation states, edge functional groups continuously adjust their charge in response to local chemical conditions, giving rise to pH-dependent surface charge regulation and buffering behavior. These findings highlight the importance of understanding the site-specific mechanisms underlying proton transfer, which are essential for interpreting surface reactivity and charge dynamics and motivate a closer examination of spontaneous proton transport under neutral conditions.

### Spontaneous Proton Transport at Neutral pH Reveals Dynamic Proton-Conducting Networks

To further elucidate the spontaneous PT observed in neutral solutions, we examined the structural origins and pathways underlying this behavior across all three nanoparticle models. Trajectory analysis revealed that the dynamic reactivity of −AlOH^–^ groups located on the B edge plays a central role. These sites consistently exhibit a tendency to acquire protons from neighboring functional groups, a behavior previously reported on similar aluminosilicate edges.^31^ In our hexagonal nanoparticle model, each B edge contains two aluminum atoms, forming two −Al­(OH)­(OH_2_) terminal motifs at junctions with two symmetrically opposite AC edges. Due to the mirror symmetry of these adjacent AC edges—one corresponding to the (110) surface and the other to the (1̅10) surface—the two resulting −AlOH^–^ sites exhibit distinct local environments and are denoted as site 1 and site 2 (Figure 3a): site 1 lies adjacent to a −SiAlO^–^ group, while site 2 neighbors a −AlOH_2_ group.

**Figure 3:**
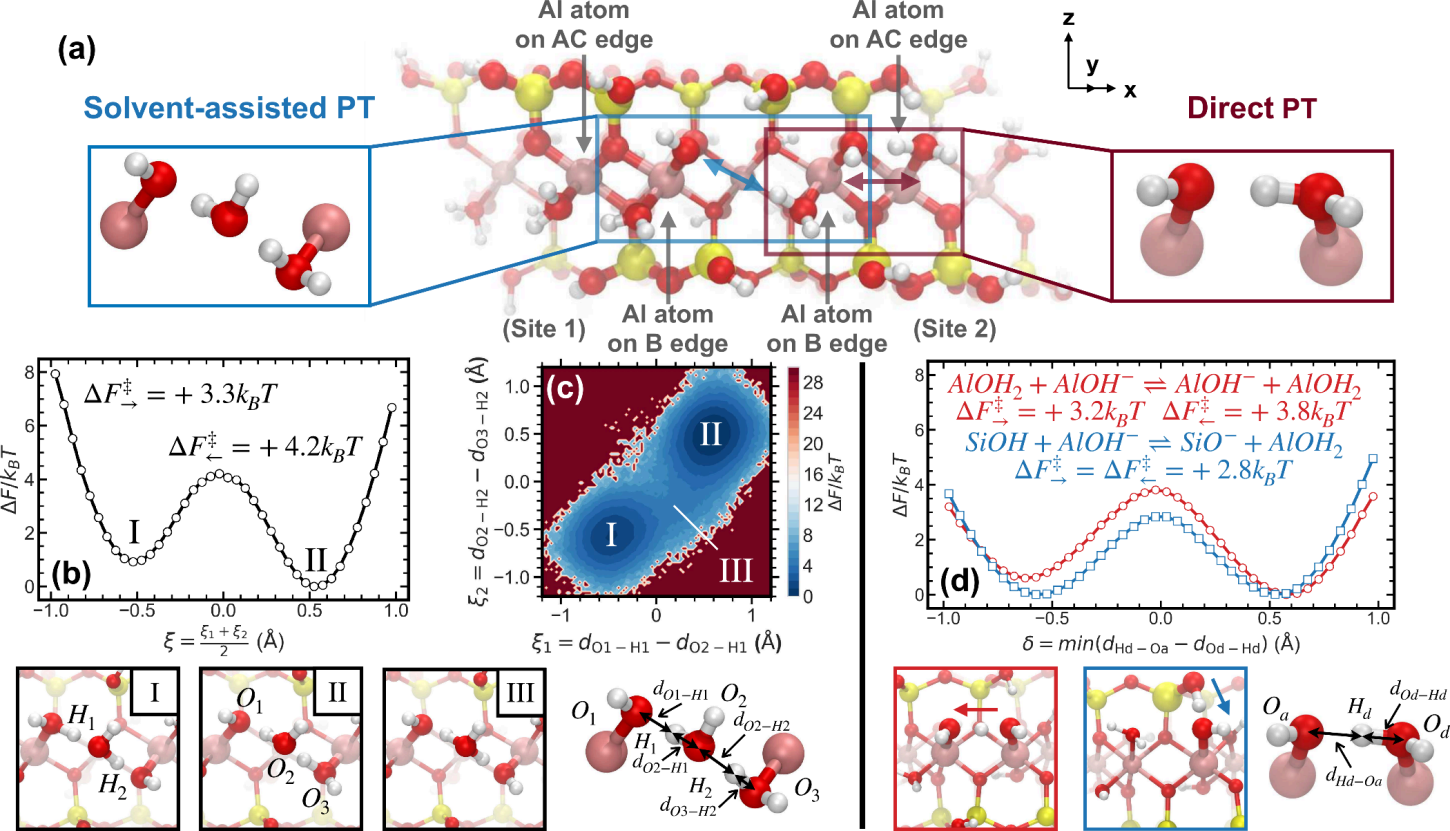
Proton transfer mechanisms occurring spontaneously on the montmorillonite nanoparticle edge surface under neutral aqueous conditions. (a) Frontal view of the B edge, showing two distinct surface environments relevant to proton transfer. Site 1 consists of an −AlOH^–^ group adjacent to a −SiAlO^–^ group and primarily undergoes solvent-assisted proton transfer. Site 2 features an −AlOH^–^ group adjacent to an −AlOH_2_ group and predominantly supports direct proton transfer. Arrows indicate the dominant PT directions at each site. Left panel (site 1, solvent-assisted PT): (b) Projected free energy profile as a function of the averaged coordinate ξ = (ξ_1_ + ξ_2_)/2. (c) Free energy surface for a solvent-assisted proton transfer process between −AlOH^–^ and −AlOH_2_ groups as a function of two collective variables, ξ_1_ = *d*_O1–H1_ – *d*_O2–H1_ and ξ_2_ = *d*_O2–H2_ – *d*_O3–H2_, in units of Å. Representative configurations (labeled I, II and III) corresponding to key points on the free energy surface are shown below panels (b) and (c). Right panel (site 2, chain-like direct PT): (d) Free energy profiles for two chain-like proton transfer reactions. The red curve with circles corresponds to the reaction AlOH_2_ + AlOH^–^⇌ AlOH^–^ + AlOH_2_, while the blue curve with squares corresponds to SiOH + AlOH^–^ ⇌ SiO^–^ + AlOH_2_. Representative structures are shown adjacent to the curves, with arrows indicating the direction of proton transfer.

These local differences give rise to distinct PT mechanisms. At site 1, the spatial separation between the −AlOH^–^ and nearby proton donors hinders direct transfer. Instead, protonation proceeds through a solvent-assisted mechanism mediated by a bridging water molecule that links the −AlOH_2_ site on the same B edge. This solvent-assisted PT pathway was observed consistently across multiple surface environments, with hundreds of events recorded over our 1.2 ns simulations, indicating its dynamic relevance and justifying further free energy analysis. To quantify the free energy barrier associated with this solvent-assisted PT, we first computed a one-dimensional proton transfer free energy landscape (PTFEL) along the averaged reaction coordinate ξ = (ξ_1_ + ξ_2_)/2, where each ξ_*i*_ reflects the position of a transferring proton relative to its donor and acceptor oxygen atoms (see Methods). Similar definitions of proton-transfer coordinates have been successfully applied in previous *ab initio* studies of multiproton transfer reactions, demonstrating that such ξ_*i*_-based coordinates reliably capture the essential features of the underlying free-energy landscape.^50^ The resulting profile shows that the free energy barrier for protonation of −AlOH^–^ (Δ*F*_←_^⧧^ = +4.2 *k*_B_ *T*) is slightly higher than that for deprotonation (Δ*F*_→_^⧧^ = +3.3 *k*_B_ *T*), indicating a mild energetic bias toward the deprotonated state (Figure 3b). To further resolve the transition pathway, we constructed a two-dimensional PTFEL using the full set of collective variables (ξ_1_, ξ_2_) (Figure 3c), highlighting three key configurations. States I and II correspond to the final and initial protonation states, while state III represents a hydronium-like intermediate along the dominant low-energy pathway. Although not labeled in the figure, hydroxide-like configurations with less favorable free energy are also accessible and are expected to contribute to the net proton fluctuations observed in Figure 2. Overall, these features suggest that PT proceeds through multiple reversible fluctuations rather than a single well-defined hopping event.

At site 2, the neighboring −AlOH_2_ group enables a direct transfer pathway. Here, protonation of the B edge −AlOH^–^ is facilitated by deprotonation of the AC edge −AlOH_2_, which in turn can receive a proton from a nearby −SiOH, forming a chain-like sequence of transfers. The corresponding PTFEL was constructed using a one-dimensional reaction coordinate δ,^51,52^ with sign assigned according to the direction of the chemical transformation (Figure 3d). The barrier for protonation of the B edge site (Δ*F*_→_^⧧^ = +3.2 *k*_B_ *T*) is lower than that of the reverse process (Δ*F*_←_^⧧^ = +3.2 *k*_B_ *T*), indicating a moderate thermodynamic preference for the protonated state. Following this step, the AC edge −AlOH_2_ group, after donating a proton, can regain their proton from a neighboring −SiOH group. The corresponding PT between −AlOH^–^ and −SiOH exhibits nearly symmetric barriers of approximately 2.8 *k*_B_ *T*, suggesting a dynamic equilibrium between these two surface groups. Both the direct PT between −AlOH^–^ and −AlOH_2_ and the subsequent transfer involving −SiOH occurred frequently, with hundreds of events recorded over the 1.2 ns simulation window. The latter pathway was observed approximately two to three times more often, reinforcing its role as the primary channel for dynamic proton exchange at the interface. This balance facilitates frequent bidirectional PTs among the B edge and AC edge groups, leading to transient surface charge fluctuations.

Together, these two pathways explain the distinct features observed in neutral conditions: the solvent-assisted transfer at site 1 accounts for the persistent net proton excess fluctuations (Figure 2a), while the direct proton hopping at site 2 explains the changes in surface group populations (Figure 2c). These mechanisms were consistently observed across all three nanoparticle models, highlighting their general significance. However, they are not the only PT routes accessible to B edge −AlOH^–^ groups. Additional transfer events involving neighboring −SiOH groups were also detected, proceeding via either direct or solvent-assisted mechanisms (see Supplementary Figure S13). These alternative pathways, while mechanistically plausible, occurred much less frequently in our simulations, with only a handful to a dozen events observed over the 1.2 ns trajectories, suggesting they play a minor role in the overall interfacial proton dynamics.

### Local Chemical Environment Modulates Proton Transfer Dynamics

While the preceding sections focused on neutral conditions, PT behaviors in acidic and basic solutions also exhibit distinct mechanistic features. Across all systems, we observed a pronounced dependence of PT frequency on the solution acidity. In acidic conditions, the number of PT events is significantly reduced compared to neutral solution, often by more than half and in some cases by an order of magnitude. This suppression arises because early protonation of key surface sites stabilizes them in their conjugate acid forms, thereby preventing further exchange. In contrast, basic conditions yield a more heterogeneous response: for some sites, PT frequency increased due to enhanced deprotonation and interaction with hydroxide ions, while for others it declined, depending sensitively on the local structure and accessibility of reactive partners.

Under basic conditions, solvent-mediated PT pathways are not limited to single bridging water molecules, but can also involve multiwater motifs. Representative snapshots illustrate such multistep proton transfer events, including transfers from −SiOH or −AlOH_2_ to neighboring −SiO^–^ groups mediated by two bridging water molecules (see Supplementary Figure S15). Such double-bridge mechanisms have been reported previously PT between −AlOH_2_ and −AlOH^–^ ^31^ and between −SiOH and −AlOH^–^.^30^ Our findings extend these mechanisms to a broader range of surface group combinations and edge contexts, demonstrating their general relevance at hydrated clay interfaces. Notably, these events do not proceed via the transfer of a localized proton along a fixed structural pathway. Instead, the transition occurs through a delocalized proton hole migrating across the hydrogen-bond network, which is characteristic of Grotthuss-like dynamics. This behavior reveals a coupling between interfacial water dynamics, transient intermediate stabilization, and long-range proton transport at mineral–water interfaces.

Beyond changes in solution pH, the local chemical environment at clay edges is further shaped by lattice composition, most notably through isomorphic substitution. Isomorphic substitution is an inherent feature of natural montmorillonite, where Mg^2+^ ions replace Al^3+^ in the octahedral layer. To assess its impact on edge reactivity, we constructed three nanoparticle models with distinct substitution patterns, enabling a systematic examination of the energetic and mechanistic consequences of substitution at different crystallographic terminations (see Supplementary Section S1).

Across acidic and basic conditions, Mg substitution generally stabilizes protonated surface species, consistent with earlier first-principles studies reporting elevated acidity constants for Mg-coordinated hydroxyl groups.^26,27^ These effects are discussed in detail in the Supplementary Section S4. In neutral solution, the effects of isomorphic substitution extend to the chain-like PT pathways at B/AC edge junctions. Specifically, when the Al atom in the AC edge of a site 2 motif is replaced by Mg, the resulting −MgOH_2_ group exhibits altered reactivity compared to its Al-based counterpart. Figure 4a presents the free energy profiles for direct PT between −SiOH and −AlOH^–^/–MgOH^–^, while Figure 4b shows PT between −AlOH_2_/–MgOH_2_ and B-edge −AlOH^–^. In both cases, Mg substitution increases the deprotonation barrier of the AC-edge donor group, thereby reducing its tendency to initiate PT. Nonetheless, the two-state character of the free energy profiles is preserved, indicating that the overall PT sequence remains feasible: the B-edge −AlOH^–^ acquires a proton from −MgOH_2_, which subsequently draws a proton from nearby −SiOH, sustaining the direct chain-like transfer. In this context, the Mg-substituted −MgOH_2_ group acts as a transient proton relay, mediating net charge migration from −AlOH^–^ to −SiO^–^. However, the elevated deprotonation barrier substantially reduces the frequency and reversibility of such events, effectively suppressing surface PT dynamics in Mg-rich environments. These findings reveal that isomorphic substitution modulates PT dynamics in multiple ways: it promotes persistent protonation at specific sites while also altering local energy landscapes to suppress frequent exchange.

**Figure 4:**
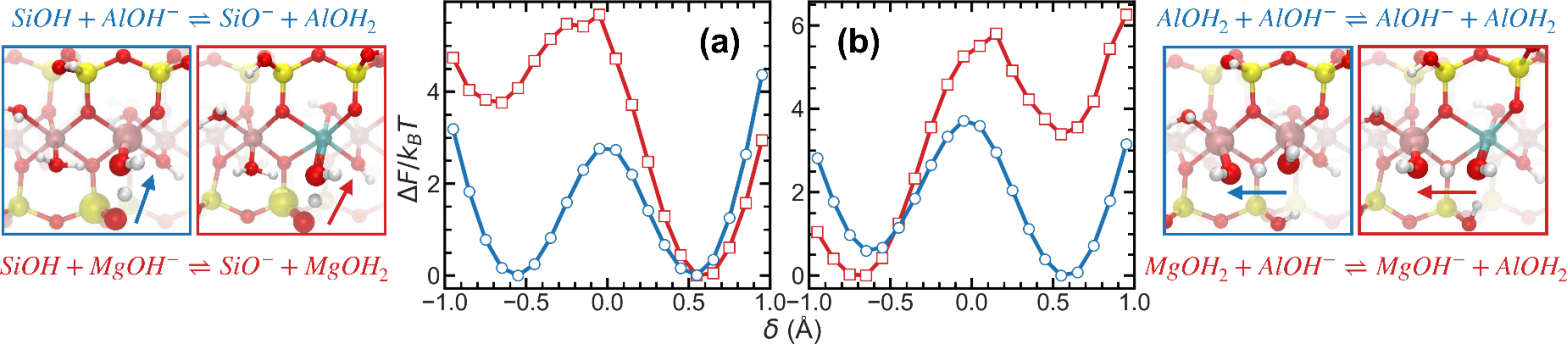
Effect of isomorphic substitution (Mg for Al) on the free energy profiles of direct proton transfer reactions in the montmorillonite nanoparticle system. Each panel compares two scenarios: red curves (highlighted by red frames) correspond to Mg-substituted sites, and blue curves (highlighted by blue frames) correspond to Al-only environments. For each case, the associated reaction equations and representative structures are shown alongside, with arrows indicating the direction of proton transfer. (a) Free energy profile for a direct proton transfer between −SiOH and −AlOH^–^/–MgOH^–^ within the site-2 region at the B/AC edge interface, under neutral conditions. (b) Free energy profile for a direct proton transfer between −AlOH_2_/–MgOH_2_ and −AlOH^–^ at the site-2 region under neutral conditions.

Overall, the combined effects of solution pH and isomorphic substitution demonstrate that proton transfer at montmorillonite edges is governed by a balance between thermodynamic stabilization and kinetic accessibility. While acidic or Mg-substituted environments favor persistent protonation, dynamic PT pathways remain accessible under environmentally relevant conditions, enabling adaptive and heterogeneous surface reactivity.

In this work, we employed MLP-MD simulations with first-principles accuracy to investigate the acid–base reactivity and PT behaviors of montmorillonite nanoparticles in aqueous environments over nanosecond time scales. Our simulations reveal that surface reactivity at montmorillonite edges is governed by proton exchange between the aqueous phase and edge functional groups, which is strongly modulated by solution pH and local chemical environment, giving rise to dynamic surface charge regulation and buffering behavior. This reactivity is expressed microscopically through the widespread occurrence of both direct and solvent-assisted PT reactions among surface functional groups. We further show that the amphoteric character of montmorillonite edge surfaces originates from the structural diversity of surface functional groups, which include both Brønsted acidic and basic moieties. The observed dynamic proton transfer activity, especially under neutral conditions, arises from the intrinsic instability of −AlOH^–^ groups on the (010) B edge, which readily engage in proton exchange processes. We further identify that basic conditions promote PT activity, while isomorphic substitution of Al by Mg increases deprotonation barriers and thereby stabilizes protonated surface states. These findings provide a molecular-level complement to macroscopic titration experiments and surface complexation models, which cannot unambiguously resolve site-specific reactivity or dynamic proton exchange. Our results show that clay edges are not static arrays of surface groups with fixed p*K*_a_ values, but dynamic, proton-conducting networks whose chemistry is tunable by both composition and environment. By explicitly simulating the interactions between edge functional groups and aqueous acid–base species, our work offers the first direct mapping of the reactive sites, mechanistic pathways, and characteristic time scales governing surface charge regulation in montmorillonite. This capability enables a more complete and predictive understanding of clay surface chemistry under realistic environmental conditions.

Our results complement previous static and short-time scale simulations by explicitly capturing the dynamic and recurrent nature of surface proton-transfer reactions, including intersite mechanisms that are difficult to access with static approaches. By identifying the influence of both surface topology and isomorphic substitution on reactivity, our study clarifies why certain sites remain inert under acidic conditions while others remain persistently active under basic conditions. The observed solvent-assisted mechanisms further highlight the importance of the surrounding hydrogen-bonding network in modulating PT barriers and enabling transient charge delocalization along clay edges. These insights support a more realistic picture of clay edge reactivity, where protons are not localized but can hop across a network of labile sites. This proton mobility is expected to play a key role in charge buffering, interfacial conductivity, and dynamic adsorption behavior in natural and engineered clay-based systems.

The simulation framework developed here can be readily extended to larger-scale clay interfaces and composite systems, enabling long-time scale tracking of surface reactivity, dissolution, and nanoparticle aggregation phenomena. A deeper understanding of surface proton dynamics will facilitate the development of predictive models for the adsorption and desorption of metal cations, organics, and pollutants on clay minerals.^23,53^ Moreover, the dynamic nature of hydrogen-bond networks and mobile protons at clay edges may provide design insights for catalytic applications involving activated clays,^54,55^ as well as for optimizing the ionic conductivity and surface charge balance of clay-based materials in energy storage devices.^56^ These findings underscore the central role of edge-resolved, proton-mediated mechanisms in shaping the broader physicochemical behaviors of clay mineral systems.

## Methods

### Machine Learning Potentials

The machine learning potential used in this work was developed within the MACE framework,^48^ which combines message passing with high body-order equivariant features to accurately capture many-body interactions. This architecture has demonstrated excellent transferability and accuracy across diverse chemical systems.^57^ Recent systematic benchmarks have demonstrated that, when trained to comparable first-principles reference data and accuracy, different modern machine-learning potential architectures yield highly consistent structural, thermodynamic, and dynamical properties, with only minor framework-dependent differences in practice.^58^ For this study, the MACE model was configured with two message-passing layers and four-body equivariant features, using a radial cutoff of 5 Å. Although explicit long-range interactions are not directly included, the receptive field of the model—defined by the product of the cutoff and the number of layers—effectively reaches 10 Å, which is sufficient to capture the relevant short- and medium-range interactions and to describe the dominant mechanisms controlling proton exchange and relaxation at the clay–water interface on the time scales investigated here.

The training data set was generated from DFT calculations using the CP2K simulation package,^59^ based on the Gaussian and plane wave (GPW) method with a high plane-wave cutoff of 1200 Ry.^60^ The revPBE functional^61,62^ combined with Grimme’s D3 dispersion correction^63^ was adopted to provide a balanced description of hydrogen-bonded liquids and silicate solids.^64^ This choice has been validated in prior studies for modeling both the structure and dynamics of water^65^ and the vibrational and mechanical properties of clay minerals,^34^ and revPBE-D3 has further been shown to perform among the most reliable functionals for liquid water even when benchmarked against higher-rung Jacob’s ladder methods that explicitly include nuclear quantum effects.^66^ Electron–ion interactions were treated using Goedecker–Teter–Hutter (GTH) pseudopotentials^67^ with element-specific basis sets: TZV2P-GTH for H, O, Na, and Cl, and DZVP-MOLOPT-SR-GTH for Al, Mg, and Si. All calculations employed periodic boundary conditions, and simulation cells were constructed to match the structural features of each system, while retaining charge neutrality.

The training data set encompassed bulk aqueous solutions, diverse montmorillonite surfaces, and interface structures under different pH conditions, capturing both structural and chemical variability (see Supplementary Section S1). The trained model achieves low root mean squared errors (RMSEs) of 0.4 meV/atom in energies and 26.2 meV/Å in forces on the training set, with similarly low values on the test set (0.5 meV/atom and 27.7 meV/Å). Further validation confirmed the model’s reliability in reproducing interfacial energetics, clay lattice structures, bulk water properties, and proton transfer free energies, including p*K*_a_ values for representative surface terminations consistent with previous AIMD studies^26^ and an accurate description of water self-dissociation in line with literature estimates^68,69^ (see Supplementary Section S2).

### Molecular Dynamics Simulations

MLP-based molecular dynamics simulations were conducted in LAMMPS^70^ under *NPT* conditions at 298 K and 1.01325 bar using the Nosé–Hoover thermostat and barostat. A time step of 0.5 fs was used, and three central oxygen atoms of each clay particle were constrained to preserve structural integrity. Simulations were performed on three montmorillonite nanoparticles, each embedded in acidic, neutral, and basic aqueous environments, resulting in nine total systems. Each trajectory was run for 1.2 ns with periodic boundary conditions in all directions, and representative configurations were extracted for further analysis, resulting in total in 10 ns for analysis.

### Proton Transfer Free Energy Landscape

PT events at montmorillonite edges were analyzed by constructing one- and two-dimensional free energy profiles from molecular dynamics trajectories. Two types of PT mechanisms were considered: direct PT between neighboring surface sites and solvent-assisted transfer mediated by bridging water molecules.

For direct PT, hydrogen bonds were first identified based on geometric criteria (O_d_–O_a_ < 3.5 Å, O_a_–O_d_–H_d_ < 30°).^71^ A reaction coordinate δ was defined for each hydrogen-bond donor as

1 $$\delta =\min\left(d_{Hd-Oa}-d_{Od-Hd}\right)$$

and used to construct free energy profiles via:

2 $$\Delta F\left(\delta \right)/k_{B}T=-\ln P\left(\delta \right)$$

where *P*(δ) is the normalized probability distribution. Signs of δ were assigned to distinguish forward and reverse reactions, as annotated in each figure.

For solvent-assisted PT, configurations involving a water molecule bridging two reactive edge sites (O_1_ and O_3_) were identified, with O_2_ denoting the central bridging oxygen. Two reaction coordinates were defined to describe the proton positions relative to their donor and acceptor oxygen atoms:

3 $$\xi_{1}=d_{O2-H1}-d_{O1-H1},,\xi_{2}=d_{O2-H2}-d_{O3-H2}$$

For one-dimensional analysis, we used the averaged coordinate ξ = (ξ_1_ + ξ_2_)/2. For two-dimensional profiles, the joint distribution *P*(ξ_1_, ξ_2_) was used to compute:

4 $$\Delta F\left(\xi_{1},\xi_{2}\right)/k_{B}T=-\ln P\left(\xi_{1},\xi_{2}\right)$$

Complete definitions, identification criteria, and sampling procedures are detailed in Supplementary Section S4.

## Supplementary Material





## Notes

Development and validation of the machine learning potential; detailed simulation results for different nanoparticle configurations; and additional data on proton transfer behaviors. (PDF)
Transparent Peer Review report available (PDF)
