# Supplementary material for: Clay Edges Are Dynamic Proton-Conducting Networks Modulated by Structure and pH
Source: J Phys Chem Lett. 2026 Feb 23;17(9):2679–88. doi: 10.1021/acs.jpclett.5c03748 (PMC12969369; doi:10.1021/acs.jpclett.5c03748)
Supplement: Supplementary file 1 [file jz5c03748_si_001.pdf]

Supporting Information: Clay Edges Are Dynamic Proton-conducting Networks Modulated by Structure and pH

Yixuan Feng,[a] Xavier R. Advincula,[b,c,d] Hongwei Fang,[a] Christoph Schran,[c,d]

[a] *State Key Laboratory of Hydro-science and Engineering, Department of Hydraulic Engineering, Tsinghua University, Beijing 100084, China.*

[b] *Yusuf Hamied Department of Chemistry, University of Cambridge, Lensfield Road, Cambridge, CB2 1EW, UK.*

[c] *Cavendish Laboratory, Department of Physics, University of Cambridge, Cambridge, CB3 0HE, UK.*

[d] *Lennard-Jones Centre, University of Cambridge, Trinity Ln, Cambridge, CB2 1TN, UK.*

E-mail: fanghw@tsinghua.edu.cn, cs2121@cam.ac.uk

Contents

1 Development of Machine Learning Potential 3
- 1.1 Montmorillonite Nanoparticles . . . 3
- 1.2 Dataset Construction . . . 4
- 1.3 DFT Calculations for Reference Data . . . 6
- 1.4 Training of the Machine Learning Potential . . . 7
- 1.5 Modelcular Dynamics Simulations . . . 7

2 Validation of Machine Learning Potentials 8
- 2.1 Validation on Interfacial Systems . . . 8
- 2.2 Validation on Pyrophyllite Supercell . . . 9
- 2.3 Validation on Bulk Water . . . 10
- 2.4 Validation on Proton Transfer Free Energies in Bulk Solution . . . 11
- 2.5 Consistency of Relative Proton Activity with Reported pK_a Trends . . . 12
- 2.6 Validation of Montmorillonite Edge Sites pK_a . . . 12

3 Acid–Base Reactivity of Different Nanoparticles 16

4 Proton Transfer Free Energy Landscape 19
- 4.1 Direct Proton Transfer . . . 19
- 4.2 Solvent-assisted Proton Transfer . . . 19
- 4.3 Proton Transfer Events at the B Edge . . . 21
- 4.4 Proton Transfer Events at the AC Edge . . . 23
- 4.5 Water-mediated Multi-step Proton Transfer Events . . . 25
- 4.6 Isomorphic substitution influence in acidic and basic solution . . . 26
- 4.7 Proton Transfer Events of $-$AlMgOH . . . 27

1 Development of Machine Learning Potential

1.1 Montmorillonite Nanoparticles

The montmorillonite structures used in this study were constructed based on experimental data,[1] incorporating isomorphic substitutions of magnesium for aluminum within the octahedral sheet. The nanoparticles were constructed to predominantly expose the (110) and (010) edge surfaces, herein referred to as the AC and B edges, respectively.[2] For clarity, these designations (AC and B) are consistently used throughout this work. The AC and B edge surfaces constitute approximately 60% and 20% of the total edge area,[3] respectively, and are considered among the most stable terminations for montmorillonite.[4–6]

To comprehensively investigate the interfacial properties between montmorillonite and aqueous solution, three distinct nanoparticle structures (denoted as Mont.1, Mont.2, and Mont.3) were developed. These structures share an identical total number of isomorphic substitutions (three Mg-for-Al substitutions), but differ in the spatial distribution of the substitution sites, which were assigned randomly. The isomorphic substitutions located within the particle interior and on the AC edges were charge-balanced by Na^+ counterions. In contrast, substitutions at the B edges were compensated by protonation of Mg atoms, forming terminal $–(OH_2)_2$ groups. For Al atoms situated at these edge sites, one $–OH_2$ and one –OH group were formed. The detailed structural formulas and compositional information of each nanoparticle model are summarized in Table S1.

Table S1: Compositional and structural characteristics of the montmorillonite nanoparticles.

**Structure**	**Isomorphic Substitution Pattern**	**Visualization**
Mont.1 $Na_2HSi_{48}(Al_{21}Mg_3)O_{96}(OH)_{72}$	$N_{\text{inner}} = 2$ $N_{B\ \text{edge}} = 1$	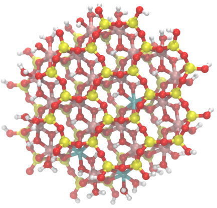
Mont.2 $Na_3Si_{48}(Al_{21}Mg_3)O_{96}(OH)_{72}$	$N_{\text{inner}} = 1$ $N_{AC\ \text{edge}} = 2$	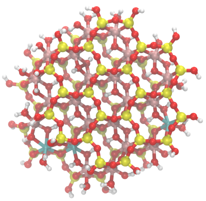
Mont.3 $Na_2HSi_{48}(Al_{21}Mg_3)O_{96}(OH)_{72}$	$N_{\text{inner}} = 1$ $N_{AC\ \text{edge}} = 1$ $N_{B\ \text{edge}} = 1$	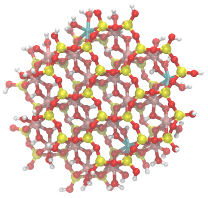

1.2 Dataset Construction

To develop a machine learning potential (MLP) capable of accurately modeling the complex montmorillonite–aqueous solution interface, the dataset must comprehensively represent a wide range of relevant chemical environments. These include bulk aqueous solutions and explicit solid–liquid interface systems. Furthermore, to ensure that the resulting MLP has sufficient generalization ability and transferability to larger-scale interfaces and various montmorillonite structures, the dataset was designed to include diverse system sizes, aqueous solutions with different pH conditions, and montmorillonite nanoparticles with different distributions of isomorphic substitutions. A detailed summary of the dataset composition is provided in Table S2.

All configurations were generated through molecular dynamics (MD) simulations performed in the NPT ensemble using the LAMMPS package[7] and starting from the MACE-MP-0 foundation model.[8] We also generated further training configurations in a reinforcement cycle using preliminary MACE models trained to the target revPBE-D3 reference to improve data set coverage. The simulations were conducted at temperatures ranging from 298 K to 400 K with a time step of 0.5 fs, generating a total trajectory length of 50 ps for each condition listed in Table S2. Temperature and pressure were controlled using Nosé–Hoover thermostat and barostat with damping parameters of 50 fs and 500 fs. For each system, 30–40 structures were randomly selected from the MD trajectories, resulting in a dataset consisting of 580 unique atomic configurations. Although the number of unique configurations is moderate, the resulting dataset includes a total of 2,417,016 force components, providing ample coverage for model training and validation.

Table S2: Details of the dataset

System	Number	Simulation details	Visualization
Bulk water	40	$N_{water} = 300$	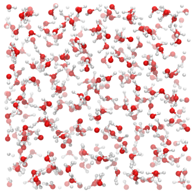
NaCl solution	30	$N_{water} = 500$<br>$N_{Na^+} = 10$<br>$N_{Cl^-} = 10$	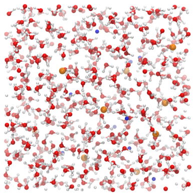
NaCl-HCl solution	30	$N_{water} = 500$<br>$N_{Na^+} = 5$<br>$N_{H3O^+} = 5$<br>$N_{Cl^-} = 10$	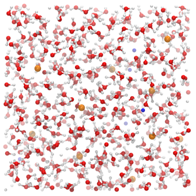

System	Number	Simulation details	Visualization
NaCl-NaOH solution	30	$N_{water} = 500$<br>$N_{Na^+} = 10$<br>$N_{OH^-} = 5$<br>$N_{Cl^-} = 5$	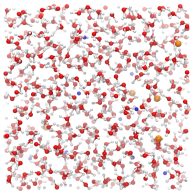
NaCl pair	30	$N_{water} = 300$<br>$N_{Na^+} = 1$<br>$N_{Cl^-} = 1$	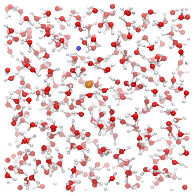
HCl pair	30	$N_{water} = 300$<br>$N_{H3O^+} = 1$<br>$N_{Cl^-} = 1$	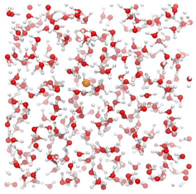
NaOH pair	30	$N_{water} = 300$<br>$N_{Na^+} = 1$<br>$N_{OH^-} = 1$	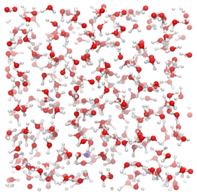
$Si_{48}Al_{24}O_{96}(OH)_{72}$–water interface	40	$N_{Mont.\ particle} = 1$<br>$N_{water} = 250$	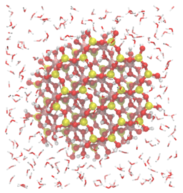
$Si_{48}Al_{24}O_{96}(OH)_{72}$–acid solution interface	40	$N_{Mont.\ particle} = 1$<br>$N_{water} = 250$<br>$N_{H3O^+} = 3$<br>$N_{Cl^-} = 3$	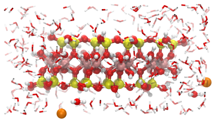
$Si_{48}Al_{24}O_{96}(OH)_{72}$–base solution interface	40	$N_{Mont.\ particle} = 1$<br>$N_{water} = 250$<br>$N_{Na^+} = 3$<br>$N_{OH^-} = 3$	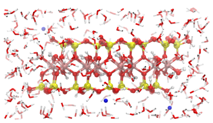

System	Number	Simulation details	Visualization
Mont.1–water interface	40	$N_{Mont.\ particle} = 1$<br>$N_{water} = 250$	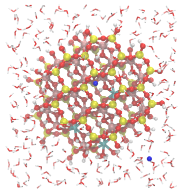
Mont.1–acid solution interface	40	$N_{Mont.\ particle} = 1$<br>$N_{water} = 250$<br>$N_{H3O^+} = 3$<br>$N_{Cl^-} = 3$	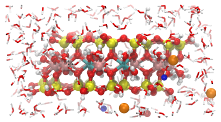
Mont.1–base solution interface	40	$N_{Mont.\ particle} = 1$<br>$N_{water} = 250$<br>$N_{Na^+} = 3$<br>$N_{OH^-} = 3$	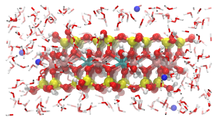
Mont.2–water interface	40	$N_{Mont.\ particle} = 1$<br>$N_{water} = 750$	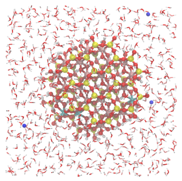
Mont.2–acid solution interface	40	$N_{Mont.\ particle} = 1$<br>$N_{water} = 750$<br>$N_{H3O^+} = 5$<br>$N_{Cl^-} = 5$	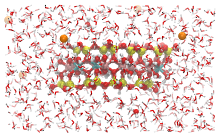
Mont.2–base solution interface	40	$N_{Mont.\ particle} = 1$<br>$N_{water} = 750$<br>$N_{Na^+} = 5$<br>$N_{OH^-} = 5$	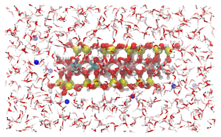

1.3 DFT Calculations for Reference Data

To generate total energies and atomic forces for training the MLP, density functional theory (DFT) calculations were performed on all configurations in the dataset using the CP2K simulation package.[9] The Gaussian and plane wave (GPW) method was employed with a plane-wave cutoff of 1200 Ry to ensure high numerical accuracy.[10] The exchange–correlation interaction was described by the revPBE functional[11,12] combined with Grimme's D3 dispersion correction.[13] This functional was chosen due to its ability to provide a balanced and reliable description of both aqueous systems and clay minerals. In particular, it yields good accuracy in modeling the structure and dynamics of water,[14] while also performing well in reproducing vibrational and structural properties of clay materials.[15] While revPBE-D3

has known limitations for isolated cation hydration, the present focus on edge reactivity at clay–water interfaces motivates its use as a consistent and computationally tractable reference for generating reactive training data, consistent with widely used clay force fields[16] and supported by our validation of clay structures, bulk water, and proton transfer free-energy profiles. Electron–ion interactions were modeled using Goedecker–Teter–Hutter (GTH) pseudopotentials,[17] along with element-specific basis sets. A triple-ζ valence basis set with polarization (TZV2P-GTH) was used for H, O, Na, and Cl atoms, while a double-ζ valence basis set from the MOLOPT-SR family (DZVP-MOLOPT-SR-GTH) was used for Al, Mg, and Si. All calculations were conducted under periodic boundary conditions, with system dimensions defined according to the specific structure of each configuration. The energy and force outputs from these DFT calculations were used as the reference data for training of the MLP.

1.4 Training of the Machine Learning Potential

The MLP employed in this study was developed using the MACE framework,[18] which combines message passing with high body-order equivariant features. This architecture has demonstrated convincing accuracy and transferability across a wide range of systems.[19] In this work, the MACE model was configured with two message-passing layers and four-body equivariant features, using a cutoff radius of 5 Å. Although explicit long-range electrostatics are not included, the effective receptive field of the model is given by the product of the cutoff distance and the number of message-passing layers, reaching approximately 10 Å. This interaction range is sufficient to capture the dominant short- and medium-range interactions between solvated protons/hydroxide ions and clay edge surfaces in the present simulation cell.

To enable accurate modeling of the complex montmorillonite–aqueous interface, the potential was trained on a dataset composed of total energies and atomic forces calculated from DFT, as described in the previous section. The training set consisted of 500 configurations randomly sampled from the full DFT dataset, while an additional 80 configurations were reserved for testing. During training, 5% of the data was used as validation set to monitor generalization performance.

1.5 Modelcular Dynamics Simulations

MLP-based MD simulations were carried out using the LAMMPS package under the NPT ensemble. A time step of 0.5 fs was employed. Temperature and pressure were maintained at 298 K and 1.01325 bar, respectively, using the Nosé–Hoover thermostat and barostat, with damping constants of 50 fs and 500 fs. To maintain structural stability, three central oxygen atoms in the clay particles were fixed throughout the simulations.

To systematically compare the behavior under different conditions, MD simulations were performed for all three montmorillonite nanoparticles (Mont.1, Mont.2, and Mont.3) in acidic, neutral, and basic aqueous environments, resulting in a total of nine systems. Each system was simulated for 1.2 ns, and configurations were sampled every 2 fs during the production runs. In the neutral systems, each nanoparticle was solvated with 750 water molecules. In acidic systems, five hydronium (H_3O^+) ions and five chloride (Cl^-) ions were added, while in basic systems, five hydroxide (OH^-) ions and five sodium (Na^+) ions were included. Periodic boundary conditions were applied in all three dimensions for every system.

2 Validation of Machine Learning Potentials

2.1 Validation on Interfacial Systems

Given the complexity of the modeled interface—comprising clay nanoparticles, aqueous solution, and the interaction region between them—we validate the MLP from three perspectives: its accuracy in predicting interfacial energies and forces, its structural reliability on clay systems, and its consistency with the structural features of bulk water. First, as shown in Figure S1, the root-mean-square errors (RMSEs) of energies and forces on the training and test datasets demonstrate that the MLP achieves high accuracy in reproducing the DFT reference data.

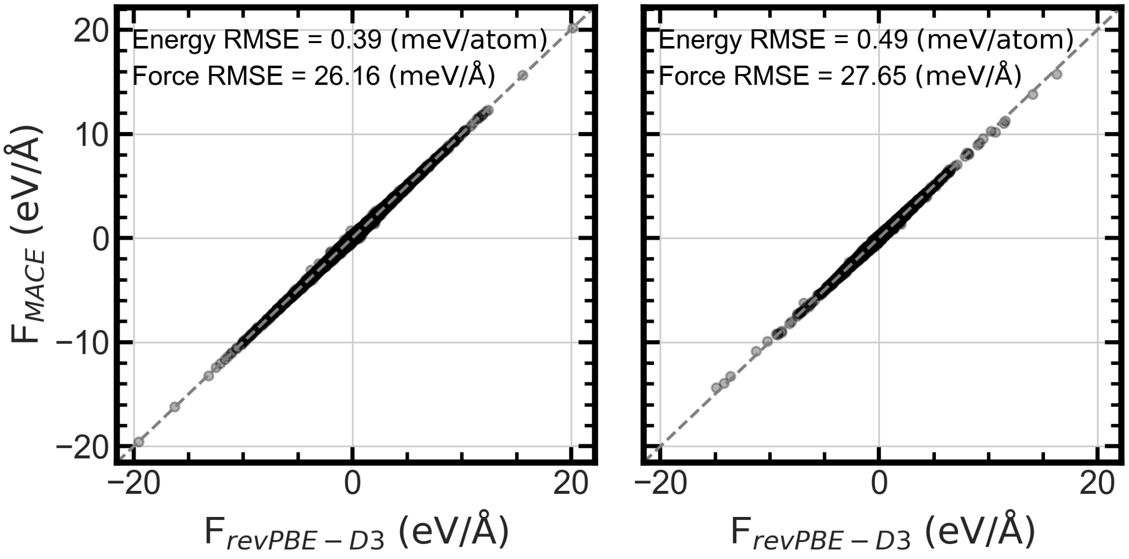

Figure S1: Correlation plots of atomic forces for the training (left) and test (right) datasets predicted by the MACE model compared to the DFT reference values. The dashed grey line indicates a perfect correlation coefficient of 1.

To further validate the model in real simulation scenarios, we extracted 45 representative snapshots from the 1.2 ns production trajectory of each interfacial system (Mont.1, Mont.2, Mont.3, each combined with different aqueous solutions), resulting in a total of 405 interfacial configurations. DFT single-point calculations were performed on these snapshots to obtain reference energies and forces. As shown in Figure S2, the resulting RMSEs between the MLP predictions and the DFT references are less than 0.5 meV/atom for energies and 30 meV/Å for forces. These results confirm that the MLP can reliably reproduce the energetics and interatomic interactions in complex montmorillonite–solution interfaces.

It is worth noting that certain systems included in this evaluation were not part of the training set. For instance, the Mont.1 system surrounded by 750 water molecules (while only a 250-water-molecule configuration was included during training), as well as all configurations involving the Mont.3 clay structure, were excluded from the training dataset. Nonetheless, the MLP achieves excellent agreement with revPBE-D3 results across these systems, further demonstrating its transferability and robustness in capturing interfacial properties across diverse clay mineral structures.

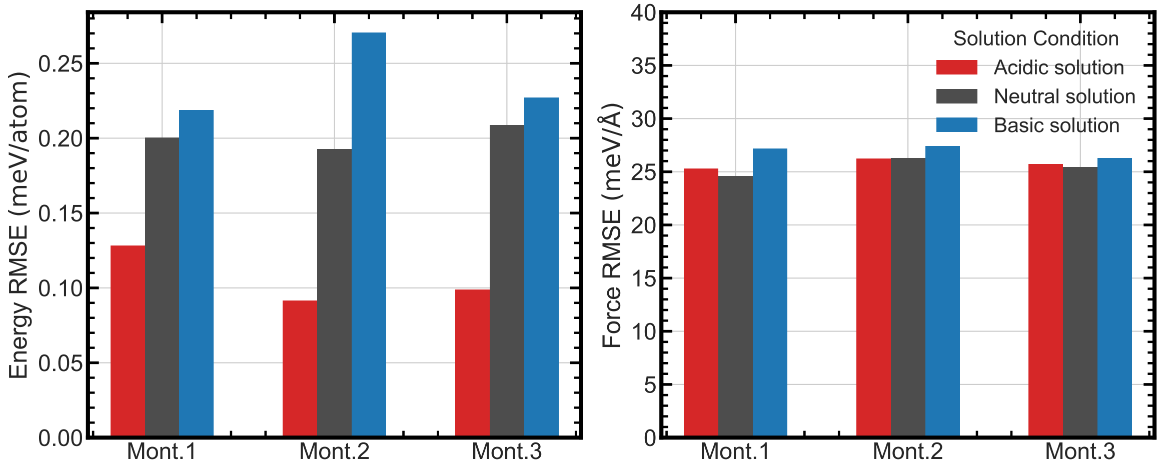

Figure S2: RMSE of energy and force predictions from the MLP compared to reference revPBE-D3 DFT results for nine interfacial systems.

2.2 Validation on Pyrophyllite Supercell

To assess the model's capability in reproducing clay mineral structures, we evaluated the relaxed cell parameters of a 2 × 2 × 2 pyrophyllite supercell (Figure S3). Pyrophyllite, like montmorillonite, is a dioctahedral 2:1 layered clay mineral with similar structural features, but it lacks isomorphic substitutions. As a result, its unit cell parameters are more uniform and well-characterized in experimental studies, whereas those of montmorillonite typically vary due to compositional heterogeneity and structural disorder.

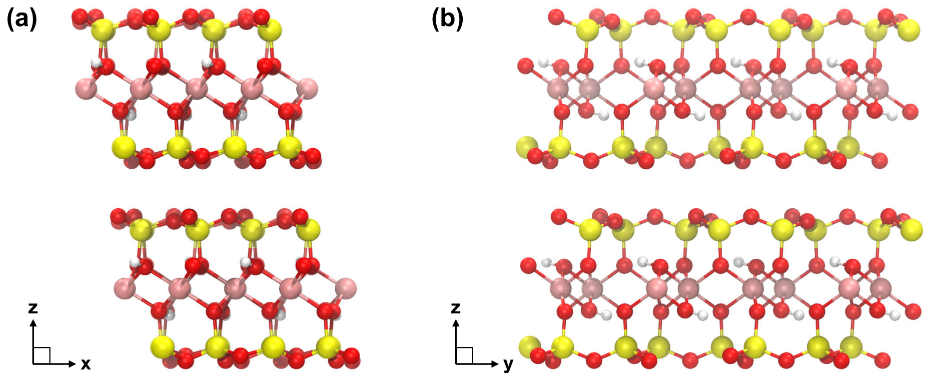

Figure S3: Snapshots of the pyrophyllite supercell (a) in the xz-plane and (b) in the yz-plane.

In this study, both DFT and MLP optimizations were initiated from the same experimental pyrophyllite structure (see Ref.[20]). The DFT calculations employed the revPBE-D3 functional to directly perform full geometry and cell optimization. In contrast, the MLP-based workflow involved an initial 100 ps NVT molecular dynamics simulation at 300 K, followed by structural relaxation to determine the final equilibrium cell parameters. As summarized in Table S3, the MLP-predicted lattice parameters show less than 1% deviation from the DFT-optimized values. Agreement with experimental data is also excellent. Minor discrepancies in certain bond lengths (greater than 1%) are attributed to the limitations of the revPBE-D3 functional rather than the MLP. These results confirm that the trained

MLP reliably reproduces the equilibrium geometry of layered clay minerals. Furthermore, the close agreement between MLP and DFT results for pyrophyllite—a structurally similar but chemically distinct system from montmorillonite—demonstrates the model's strong transferability across different types of dioctahedral 2:1 clay minerals. This highlights the potential of the MLP to generalize well to related clay systems beyond those explicitly included in the training set.

Table S3: Comparison of experimental, DFT, and MACE optimized structures, including cell parameters (a, b, c, α, β, γ, volume) and representative bond lengths.

	EXP†	revPBE - D3	MACE		
	Value	Value	Value	Error$_{DFT}$ (%)	Error$_{exp.}$ (%)
a (Å)	5.160	5.112	5.142	0.593	0.340
b (Å)	8.966	8.873	8.940	0.754	0.288
c (Å)	9.347	9.385	9.326	0.620	0.220
α (deg)	91.180	90.831	91.068	0.260	0.123
β (deg)	100.460	100.931	100.550	0.377	0.090
γ (deg)	89.640	89.849	89.817	0.036	0.197
Volume (Å^3)	425.160	417.929	421.455	0.844	0.871
Si-O_b* (Å)	1.612	1.625	1.626	0.016	0.850
Si–O_a* (Å)	1.633	1.649	1.650	0.034	1.033
Al–O_a* (Å)	1.915	1.930	1.933	0.150	0.923
Al–OH (Å)	1.889	1.887	1.890	0.125	0.039
O–H (Å)	0.935	0.966	0.965	0.061	3.261

† Experimental data are taken from Ref.[20]

* O_a and O_b denote apical and basal oxygen atoms, respectively.

2.3 Validation on Bulk Water

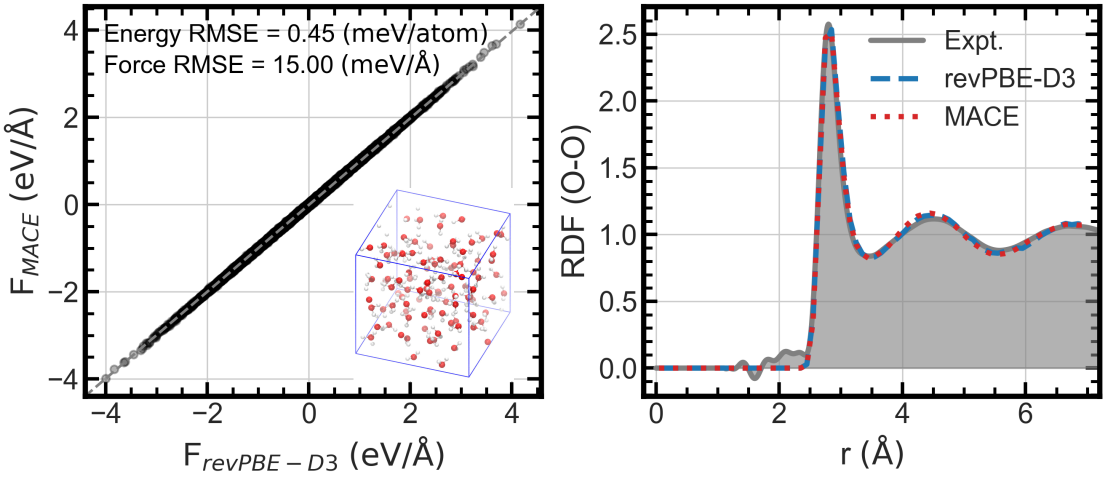

Figure S4: Correlation plot of atomic forces (left) and radial distribution function (RDF) comparison (right) for the bulk water system. The left panel shows the correlation between forces predicted by the MACE model and those from DFT calculations. The right panel compares the oxygen–oxygen RDFs computed from MACE (red dotted line), DFT (blue dashed line), and experimental measurements (gray solid line).

To evaluate the MLP's ability to capture the structural and energetic properties of liquid water,

we performed additional tests on a bulk water system. A DFT-based *ab initio* molecular dynamics (AIMD) simulation was carried out using CP2K on a system of 100 water molecules at 298 K in the NVT ensemble, producing a 10 ps trajectory. From this trajectory, 30 representative snapshots were selected, and both DFT and MLP energies and forces were evaluated. As shown in Figure S4, the RMSEs for energy and force are approximately 0.5 meV/atom and 15 meV/Å, respectively, indicating a high level of agreement between the MLP and the DFT reference.

To further validate the structural accuracy of the MLP, we computed the oxygen–oxygen radial distribution function (RDF) from the MLP simulation and compared it with that from AIMD and experimental data,[21] as shown in Figure S4. The RDFs show excellent agreement across all three systems, indicating that the MLP can reliably reproduce the structural characteristics of liquid water.

2.4 Validation on Proton Transfer Free Energies in Bulk Solution

To further evaluate the ability of the MLP to reproduce proton transfer free energetics in condensed-phase environments, we conducted additional benchmark calculations on bulk aqueous systems exhibiting either excess-proton or proton-deficient character. For each case, reference AIMD simulations were performed using the revPBE-D3 functional at 300 K, and corresponding MLP-based simulations were carried out under identical conditions. Proton transfer free energy profiles were computed for both types of defects using the same reaction-coordinate definitions employed for the direct proton-transfer mechanism in the main text, i.e., the proton-transfer coordinate δ (see Section 4.1). As shown in Figure S5, the MACE potential accurately reproduces the AIMD free-energy barriers for proton transfer in both excess-proton and proton-deficient environments, with excellent agreement across the entire reaction coordinate. This level of accuracy is fully consistent with previous studies of proton transfer in aqueous systems,[21,22] demonstrating that the MACE model reliably captures both structural and energetic aspects of proton mobility in bulk water.

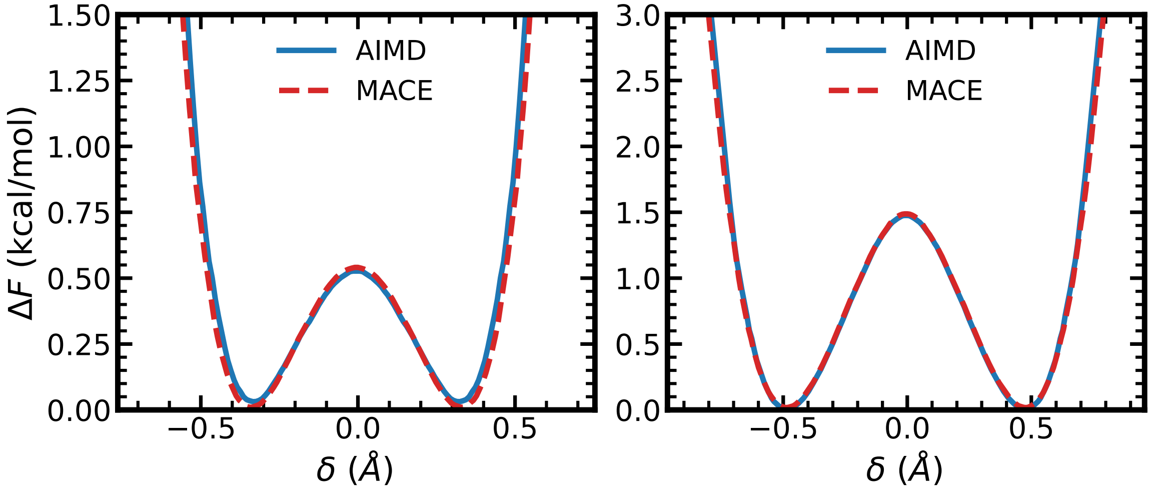

Figure S5: Proton-transfer free energy profiles for aqueous protonic defects in bulk solution. Left: proton transfer between a hydronium ion and a neighboring water molecule. Right: proton transfer between a hydroxide ion and a neighboring water molecule. In both cases, the AIMD reference free-energy curve is shown as a blue solid line, while the MACE prediction is shown as a red dashed line.

2.5 Consistency of Relative Proton Activity with Reported pK_a Trends

Acid–base properties of surface functional groups, and in particular their intrinsic pK_a values, provide a stringent benchmark for atomistic models of reactive mineral–water interfaces. Here, we examine whether relative trends in surface proton activity inferred from our neutral-water simulations are consistent with pK_a values reported in prior *ab initio* studies.

In principle, under conditions of known pH (here nominally pH $\approx$ 7), a Henderson–Hasselbalch-type relation may be used to estimate pK_a values from the relative populations of protonated and deprotonated states,

$$\mathrm{p}K_a = \mathrm{pH} - \log_{10}\left(\frac{P_{\mathrm{deprot}}}{P_{\mathrm{prot}}}\right), \tag{1}$$

where P_{prot} and P_{deprot} denote the fraction of time a given surface oxygen is found in protonated and deprotonated states, respectively.

However, an important methodological limitation arises in the context of neutral-water simulations. At pH $\approx$ 7, many surface sites remain effectively permanently protonated over the accessible simulation timescales. For such sites, population ratios do not permit the extraction of a meaningful numerical pK_a value; at best, lower bounds can be inferred. To make this distinction explicit, we therefore introduce a conservative criterion and classify a site as "active" only if its deprotonated population exceeds 0.01, corresponding to an effective p$K_a \approx 9$ at pH $\approx$ 7.

Using this criterion, we analyzed the fraction of active sites for different surface functional groups across the three nanoparticle models. Averaged over all systems, the fraction of active sites follows the ordering

$$\text{B-edge Al(OH)}_2 > \text{AC-edge Al(OH)}_2 > \text{SiOH} > \text{Mg(OH)}_2, \tag{2}$$

with approximate active fractions of 69%, 23%, 10%, and 0%, respectively.

Importantly, this ordering is fully consistent with intrinsic pK_a values reported in previous AIMD studies (p$K_a \approx$ 3.1 for B-edge $Al(OH)_2$, 5.5 for AC-edge $Al(OH)_2$, ∼7.0 for SiOH, and ∼13.2 for $Mg(OH)_2$).[23,24] Although absolute pK_a values cannot be directly extracted from neutral-water simulations, the relative proton activity of different surface groups inferred here is therefore in qualitative agreement with established first-principles results.

2.6 Validation of Montmorillonite Edge Sites pK_a

To further assess whether the machine learning potential developed in this work can faithfully describe interfacial acid–base chemistry, we compute pK_a values for representative edge surface groups of montmorillonite and compare them directly with values reported in previous AIMD studies.[23] To this end, pK_a values are obtained from free-energy profiles describing the deprotonation of different surface groups, constructed using umbrella sampling. To limit the structural heterogeneity associated with the full nanoparticle model and to enable direct comparison with previous AIMD calculations, we adopt a simplified slab representation exposing the (010) edge with the relevant surface terminations, as shown in Figure S6.

The deprotonation free-energy profiles are constructed by biasing a collective variable that quantifies proton coordination to a selected surface oxygen atom, O^*. Specifically, within each umbrella window, sampling is performed by varying the coordination number of O^* with respect to all hydrogen atoms in the simulation cell, as defined below:

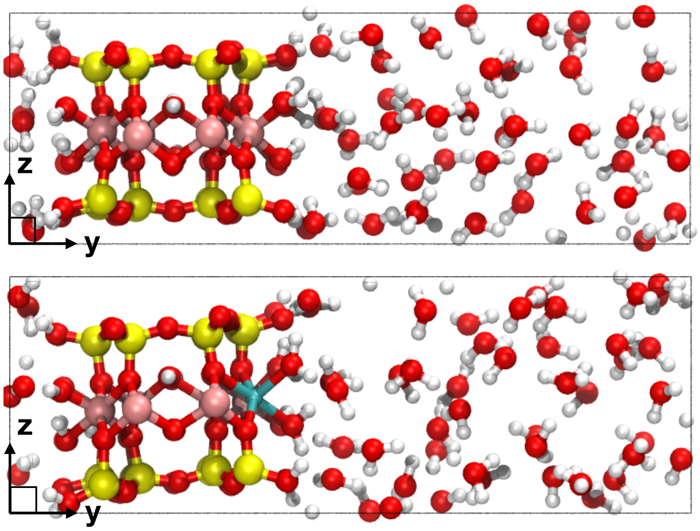

Figure S6: Slab models used for the pK_a calculations. The top panel shows the system without isomorphic substitution, while the bottom panel includes Mg-for-Al isomorphic substitution in the octahedral sheet. Atom colors are as follows: O (red), H (white), Si (yellow), Al (pink), and Mg (cyan).

$$n_{O^*-H} = \sum_{i=1}^{N} \frac{1-(r_i/R_0)^{12}}{1-(r_i/R_0)^{24}} \tag{3}$$

where the sum runs over all hydrogen atoms in the simulation cell, r_i is the distance between hydrogen i and O*, and R_0 is a characteristic switching distance fixed at 1.38 Å. Sampling along this reaction coordinate is enforced by restraining n_{O^*-H} around a target value n'_H using a harmonic bias with a force constant of 500 kcal mol^{-1} per squared coordination unit. The resulting free-energy profiles are reconstructed by umbrella integration,[25] and the pK_a values are obtained using the following expression,

$$pK_a = \frac{\Delta F_{site}}{RT \cdot \ln(10)} + \left(14 - \frac{\Delta F_{H_2O}}{RT \cdot \ln(10)}\right) \tag{4}$$

where ΔF_{site} is the free-energy difference between the protonated and deprotonated states of the surface site, R is the ideal gas constant, T is the temperature (here, 300 K), and ΔF_{H_2O} denotes the free energy associated with water self-dissociation.

Table S4: pK_a values of edge surface groups of montmorillonite.

	≡Si–OH	≡Al–OH_2OH_2	≡Mg–OH_2OH_2
pK_a (Ref. 23)	7.0 ± 0.7	3.1 ± 0.5	13.2 ± 0.5
pK_a (MLP)	7.4 ± 0.5	2.7 ± 0.3	10.2 ± 0.3

The deprotonation free-energy profiles obtained for the ≡Si–OH, ≡Al–OH_2–OH_2, and ≡Mg–OH_2–OH_2 surface groups are presented in Figure S7, together with a quantitative comparison to available AIMD results in Table S4. Across all surface terminations, we observe close agreement with previous AIMD estimates, supporting the ability of the machine learning potential developed in this work to reliably describe acid–base reactions at mineral–water interfaces. While these pK_a values agree well with AIMD benchmarks, they are derived from finite-system free-energy differences along a chosen collective variable and referenced to water self-dissociation, so they remain sensitive to the reaction coordinate

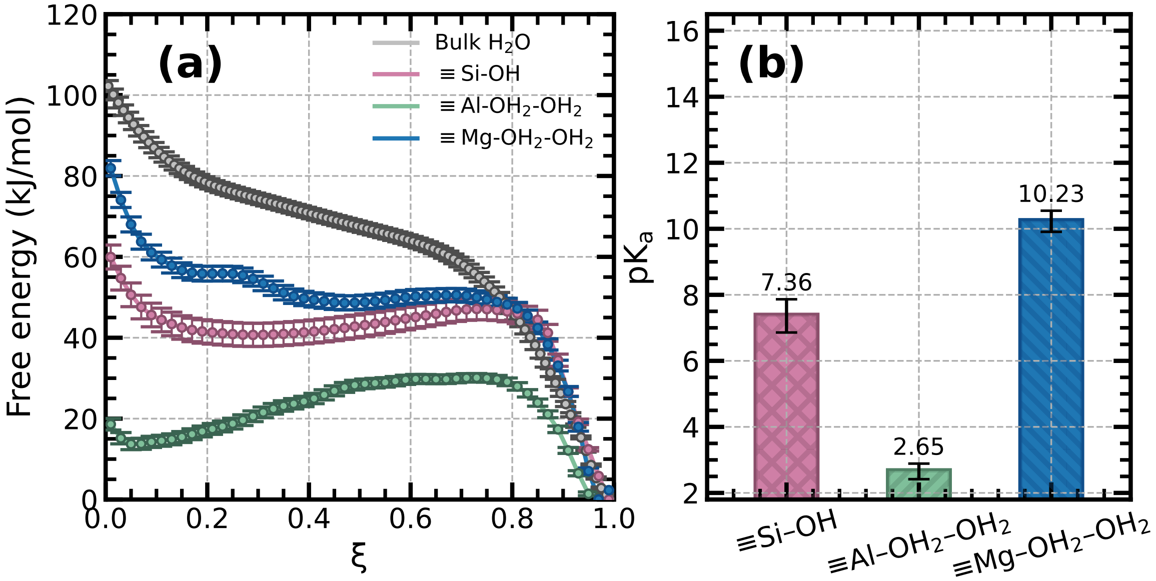

Figure S7: (a) Free-energy profiles for the deprotonation of the ≡Si–OH, ≡Al–OH_2–OH_2, and ≡Mg–OH_2–OH_2 surface groups, together with the reference water self-dissociation reaction. The reaction coordinate ξ corresponds to the coordination number $n_{\mathrm{O}^*-\mathrm{H}}$ for the ≡Si–OH group, and to $n_{\mathrm{O}^*-\mathrm{H}} - 1$ for the ≡Al–OH_2–OH_2, ≡Mg–OH_2–OH_2, and bulk H_2O reaction, allowing all profiles to be displayed on a common axis. (b) pK_a values obtained from the free-energy differences using Eq. 4. All free-energy profiles were obtained from umbrella-sampling simulations using umbrella integration. For each surface group, ten umbrella windows were employed, with simulation lengths of 250 ps per window. The reference water self-dissociation reaction was computed using 30 umbrella windows at 300 K.

definition and any residual standard-state corrections implicit in the slab geometry. We estimate that these methodological choices introduce an additional uncertainty of order 1 pH unit, comparable to typical variations between different pK_a protocols and the underlying electronic-structure theory for mineral–water interfaces. In particular, whereas earlier AIMD studies primarily employed the vertical energy gap method,[23] the present work relies on umbrella sampling to avoid the use of dummy particles important to be compatible with the current MLP framework.

When computing the pK_a values of the ≡Si–OH, ≡Al–OH_2–OH_2, and ≡Mg-OH_2-OH_2 surface groups, it is important to note that all values are referenced to the deprotonation free energy of hydronium, corresponding to the water self-dissociation reaction, following established practice in previous studies.[23,26,27] The free-energy profile for water self-dissociation is shown in grey in Figure S7 and is itself in excellent agreement with earlier AIMD results.[28,29] Using the relation $p\mathrm{K}_\mathrm{w} = \Delta F^{\ddagger}/(RT \cdot \ln 10)$, where $\Delta F^{\ddagger}$ is the free energy barrier between reactant and product states, we obtain a pK_w of 13.9 ± 0.1 at 300 K. As a further validation of the MLP, we also examined its ability to reproduce the temperature dependence of water self-dissociation. As shown in Figure S8, free-energy barriers obtained using the MACE potential closely follow the corresponding AIMD results across the full temperature range considered.[28] Moreover, the enthalpic and entropic contributions extracted from the temperature dependence of the barriers are in excellent agreement between the two approaches, indicating that the MLP captures not only accurate free-energy barriers but also the underlying thermodynamic balance governing the self-dissociation process.

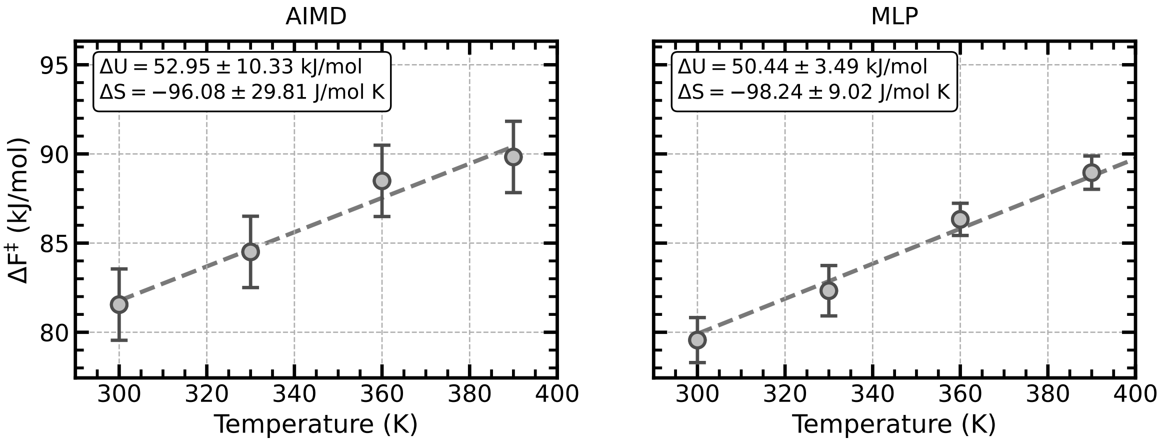

Figure S8: Comparison of temperature-dependent free energy barriers for water self-dissociation computed using AIMD[28] and the MLP developed in this work. The free energy barrier between reactant and product states, $\Delta F^{\ddagger}$, is shown as a function of temperature T for AIMD (left) and MACE (right), and fitted using the relation $\Delta F = \Delta U - T\Delta S$ to extract the enthalpic and entropic contributions. The close agreement between the two methods highlights the accuracy of the MLP in describing the dissociation process

.

3 Acid–Base Reactivity of Different Nanoparticles

In the main text, Mont.2 was selected as a representative structure to demonstrate the acid–base reactivity of montmorillonite nanoparticles in aqueous environments. Here, we extend the analysis to include Mont.1 and Mont.3, providing a comparative view of the interfacial protonation and deprotonation behaviors under acidic, neutral, and basic conditions. Figure S9 shows the time evolution of the net proton excess in the solution phase—defined as the difference between the number of hydronium ions and hydroxide ions ($H_3O^+ - OH^-$)—over a 1.2 ns molecular dynamics trajectory, along with the corresponding distribution during the final 200 ps. All three montmorillonite systems exhibit amphoteric behavior, characterized by net proton uptake under acidic conditions and net proton release under basic conditions. Notably, deprotonation in basic solution proceeds much faster than protonation in acidic environments. This is reflected in the times required for five hydroxide ions to react in the basic solution: 182 ps for Mont.1, 235 ps for Mont.2, and 121 ps for Mont.3. In contrast, the times required for five hydronium ions to react in acidic solution are significantly longer: over 1.2 ns for Mont.1, 1019 ps for Mont.2, and 634 ps for Mont.3. Interestingly, in Mont.1 under acidic conditions, the net proton excess first decreases and then increases between 730 ps and 1000 ps, indicating a transient protonation and subsequent deprotonation of a $-SiAlO^-$ group.

Figure S10 presents the relative changes in surface functional group populations during the final 200 ps of the simulation, normalized to their initial values. In acidic solution, the patterns of protonation vary significantly among the three montmorillonite structures. For Mont.1, two $-AlOH^-$ groups are protonated to $-AlOH_2$, and one $-SiAlO^-$ is protonated to $-SiAlOH$, while two hydronium ions remain unreacted in solution. In Mont.2, two $-SiMgO^-$ groups are protonated to $-SiMgOH$, one $-AlMgOH$ group becomes $-AlMgOH_2{}^+$, and four $-AlOH^-$ groups are protonated to $-AlOH_2$, including two that receive protons transferred from neighboring $-SiOH$ groups. Mont.3 shows protonation of four $-AlOH^-$ to $-AlOH_2$ and one $-SiMgO^-$ to $-SiMgOH$. In basic solution, the dominant deprotonation pathway involves $-SiOH$ groups. In Mont.2, protons are also transferred from $-SiOH$ to $-AlOH^-$, resulting in the formation of $-AlOH_2$; whereas in Mont.1 and Mont.3, $-AlOH_2$ groups undergo partial deprotonation to $-AlOH^-$. Under neutral conditions, proton transfer between $-SiOH$ and $-AlOH^-$ is observed across all three structures, highlighting the dynamic equilibrium of surface groups.

Overall, the spatial distribution of isomorphic substitutions in montmorillonite significantly influences both the number and type of reactive sites, particularly under acidic conditions. The observations from Mont.2 suggest that its specific isomorphic substitution pattern may promote either enhanced deprotonation of $-SiOH$ groups or increased proton affinity of neighboring $-AlOH-$ sites, thereby facilitating interfacial proton redistribution. These findings highlight both the common amphoteric nature of montmorillonite surfaces across different substitution patterns, as well as the subtle structural differences that modulate the extent and dynamics of acid–base reactivity at the interface.

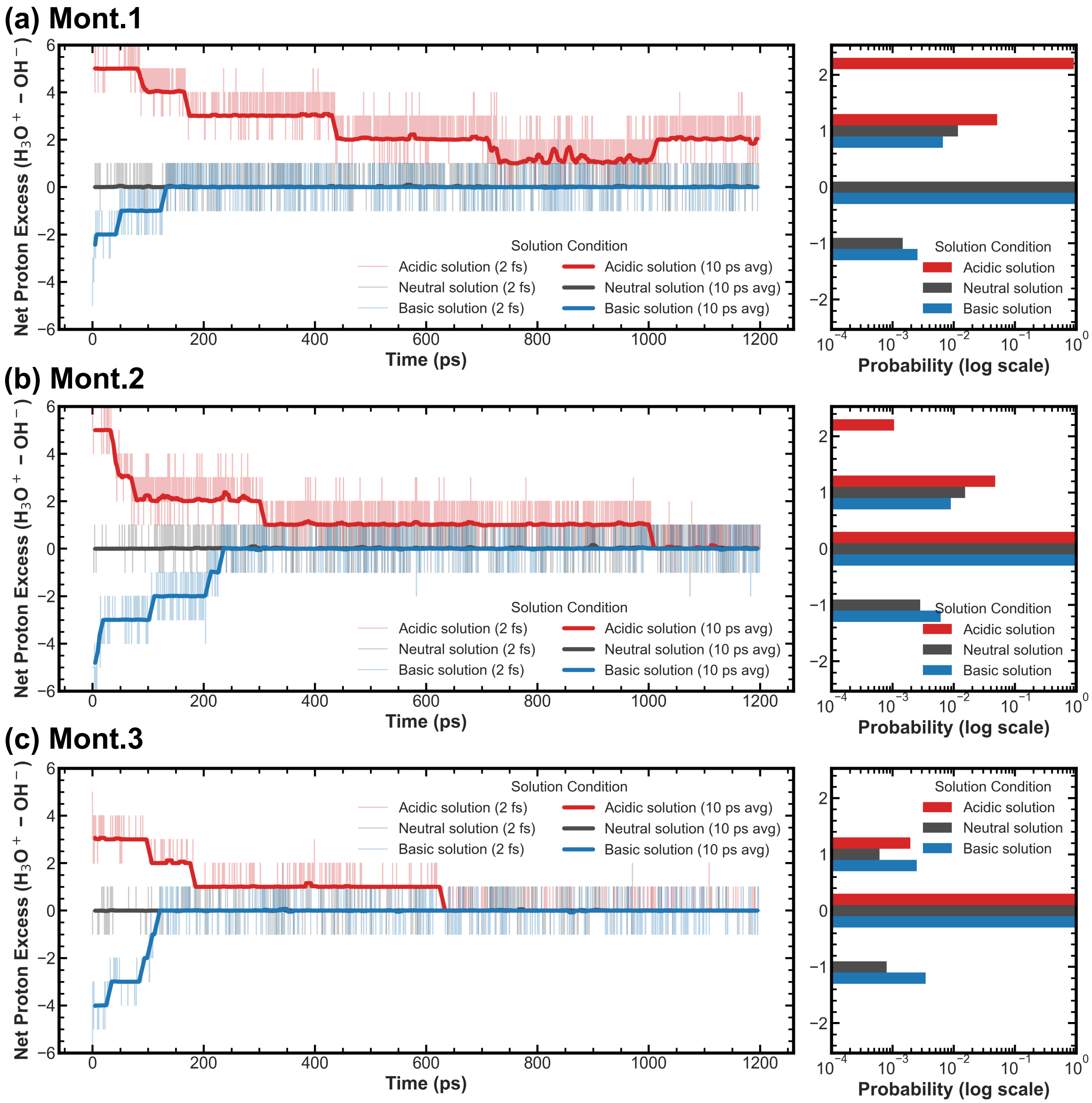

Figure S9: Time evolution and statistical distribution of the net proton excess in the aqueous phase for three montmorillonite systems. (a) Mont.1, (b) Mont.2, and (c) Mont.3. In each panel, the left subfigure shows the time evolution of the net proton excess (defined as the number of hydronium ions minus hydroxide ions) over a 1200 ps molecular dynamics trajectory. Transparent curves represent raw data sampled every 2 fs, while solid lines correspond to time-averaged values over a 10 ps window. The right subfigure presents the probability distribution of the net proton excess during the final 200 ps of the simulation. Colors represent different solution pH conditions: red for acidic, black for neutral, and blue for basic environments.

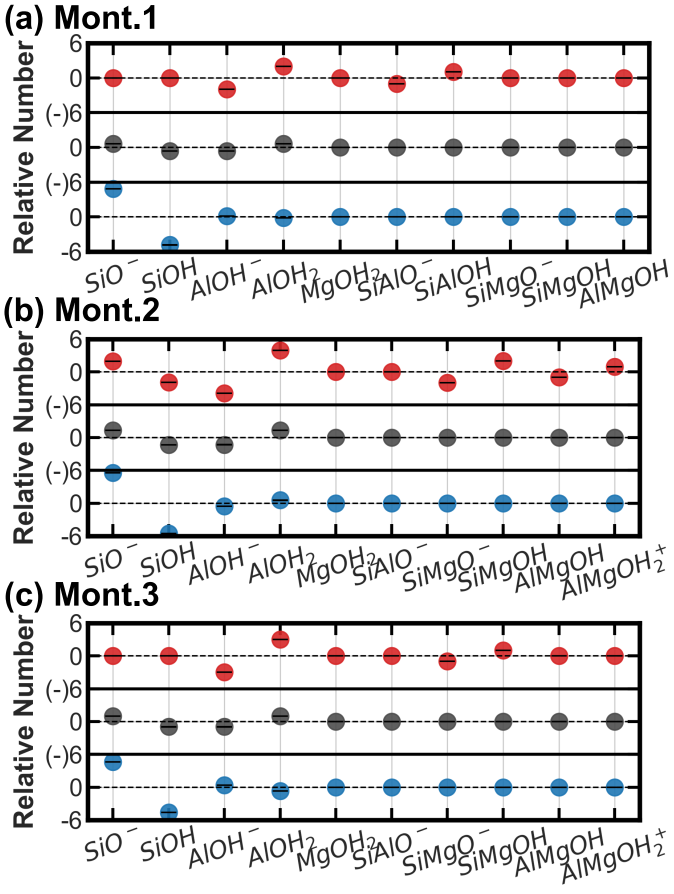

Figure S10: Relative abundance of surface functional groups on montmorillonite edges under different pH conditions. Panels (a), (b), and (c) correspond to Mont.1, Mont.2, and Mont.3, respectively. Each point represents the relative population of specific surface functional groups during the final 200 ps of a 1.2 ns molecular dynamics trajectory, normalized by their initial populations. Colors denote the solution pH: red for acidic, black for neutral, and blue for basic environments.

4 Proton Transfer Free Energy Landscape

4.1 Direct Proton Transfer

To construct the proton transfer free energy landscape (PTFEL) for direct proton transfer (PT) between surface groups on the nanoparticle edge, each hydrogen atom was first assigned to its nearest oxygen atom based on interatomic distance. Hydrogen bonds were then identified using geometric criteria: the distance Od–Oa was required to be less than 3.5 Å, and the angle Oa–Od–H_d was constrained to be smaller than 30°,[30] where the subscripts d and a denote the hydrogen-bond donor and acceptor oxygen atoms, respectively.

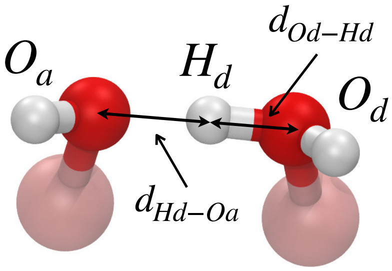

Figure S11: Schematic illustration of the variables used to describe a direct proton transfer reaction between an $-AlOH^-$ group and a neighboring $-AlOH_2$ group.

For each hydrogen-bond donor oxygen O_d, all candidate acceptors O_a were evaluated, and a PT coordinate was defined as:

$$\Delta = d_{\mathrm{Hd-Oa}} - d_{\mathrm{Od-Hd}}$$

The pair with the minimum value of Δ for each donor oxygen was identified and denoted as $\delta = \Delta_{\min}$, representing the most probable PT event for that donor. These δ values were used as the reaction coordinates for constructing the free energy profiles.

Although δ is always computed as a positive quantity by definition, its sign was reassigned based on the direction of the underlying PT process. In the plotted PTFELs, negative values of δ correspond to the forward direction of the reaction, while positive values represent the reverse direction. The corresponding chemical equations are explicitly labeled in each PTFEL figure.

The values of δ were binned (with a bin width of 0.05 or 0.1 Å), and the probability distribution $P(\delta)$ was estimated from histogram counts. The free energy profile was then computed as:

$$\Delta F(\delta)/k_B T = -\ln P(\delta)$$

where a small constant was added to avoid numerical divergence from ln(0). The resulting $\Delta F(\delta)/k_B T$ was shifted so that its minimum value is zero.

4.2 Solvent-assisted Proton Transfer

To investigate the free energy landscape of solvent-assisted PT events, we identified representative configurations in which a water molecule bridges two reactive surface oxygen atoms (O_1 and O_3) on the nanoparticle edge. For each simulation frame, the nearest oxygen atom to both O_1 and O_3 was labeled O_2.

Following the same procedure as for direct PT, hydrogen atoms were assigned to their nearest

oxygen atoms, and hydrogen bonds were identified using geometric criteria. Candidate hydrogens involved in the transfer were determined as follows: H_1 was defined as the hydrogen shared between O_1 and O_2, and H_2 as that between O_2 and O_3. Each hydrogen was chosen to minimize the distance mismatch between the two neighboring oxygens and further required to correspond to the shortest hydrogen bond for O_1 and O_3, respectively.

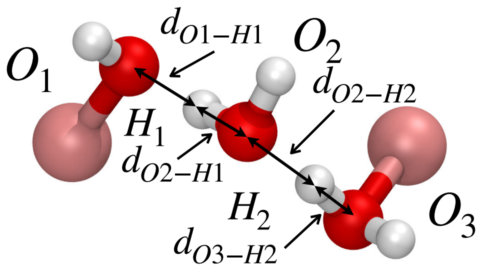

Figure S12: Schematic illustration of the collective variables used to describe a solvent-assisted proton transfer reaction between an $-AlOH^-$ group and a neighboring $-AlOH_2$ group. Two protons (H_1 and H_2) bridge three oxygen atoms (O_1, O_2, and O_3) via hydrogen bonding.

For accepted frames, donor–acceptor distances (O_1–H_1, O_2–H_1, O_2–H_2, O_3–H_2) were calculated (Figure S12). Two reaction coordinates were then defined to represent the proton positions relative to their donor and acceptor oxygen atoms:

$$\xi_1 = d_{\mathrm{O2-H1}} - d_{\mathrm{O1-H1}}, \quad \xi_2 = d_{\mathrm{O2-H2}} - d_{\mathrm{O3-H2}}$$

For one-dimensional analysis, the mean coordinate

$$\xi = \frac{1}{2}(\xi_1 + \xi_2)$$

was used, and its distribution was converted into a free energy profile by:

$$\Delta F(\xi)/k_B T = -\ln P(\xi)$$

The resulting profile was shifted so that its minimum energy was set to zero.

For two-dimensional free energy landscapes, joint histograms of (ξ_1, ξ_2) were constructed, and the corresponding free energy surface was computed as:

$$\Delta F(\xi_1, \xi_2)/k_B T = -\ln P(\xi_1, \xi_2)$$

where $P(\xi_1, \xi_2)$ denotes the normalized two-dimensional probability density.

4.3 Proton Transfer Events at the B Edge

While the main text focused on a representative PT process at the B edge of montmorillonite in neutral solution, additional distinct pathways were identified across different interfacial environments. As shown in Figure. S13, we observed diverse PT events involving $-$SiOH and $-$AlOH$^-$ groups under both neutral and basic conditions, further highlighting the dynamic reactivity of the B edge.

Pathway 1 involves a solvent-assisted proton transfer between a $-$SiOH group and a neighboring $-$AlOH$^-$ site located at site 2 of the B edge, observed under neutral conditions. Although the relatively low number of transfer events in the trajectory limits the extraction of a well-defined one-dimensional free energy profile, the two-dimensional PTFEL in Figure S13a reveals that the free energy minimum corresponds to the protonated $-$AlOH$_2$ state, indicating a weak preference for protonation. For comparison, the direct proton transfer from an adjacent $-$AlOH$_2$ group at the AC edge (discussed in Section B of the main text) has a lower barrier, making this solvent-assisted pathway energetically less favorable, but still mechanistically accessible within the simulated timescale.

Pathway 2 describes a direct PT between a $-$SiOH group and an adjacent $-$AlOH$^-$ site of the B edge, observed in neutral solution. The forward barrier for $-$AlOH$^-$ protonation ($\Delta F^{\ddagger}_{\rightarrow} = 7.7\, k_B T$) is significantly larger than the reverse barrier ($\Delta F^{\ddagger}_{\leftarrow} = 4.7\, k_B T$), indicating that although the reaction can occur, it is less favorable and thus sampled less frequently (Figure S13b, right). In basic solution, a solvent-assisted pathway was identified at the same site. (Figure S13b, left) Due to the relatively low number of PT events and the influence of other stable, non-reactive configurations, a clear one-dimensional free energy profile could not be extracted from the two-dimensional surface, preventing direct comparison of activation barriers. Nevertheless, its comparable occurrence frequency indicates that this pathway can also proceed via solvent-assisted proton transfer.

Pathway 3 involves PT between a $-$SiOH group located near the junction of the AC and B edges and a neighboring $-$AlOH$^-$ site at site 1. In basic solution, both direct and solvent-assisted pathways were observed to occur reversibly between the same pair of functional groups. (Figure S13c) Notably, the free energy profiles differ: the direct PT favors the formation of $-$AlOH$_2$ and $-$SiO$^-$, while the solvent-assisted PT favors $-$AlOH$^-$ and $-$SiOH. This observation highlights that not only the PT barriers but also the relative stability of the resulting protonation states can be influenced by the mechanism—direct versus solvent-assisted.

Together, these findings underscore the complexity of edge-site acid–base chemistry in montmorillonite and demonstrate that both local structure and interfacial environment play a critical role in determining reaction pathways and energetics.

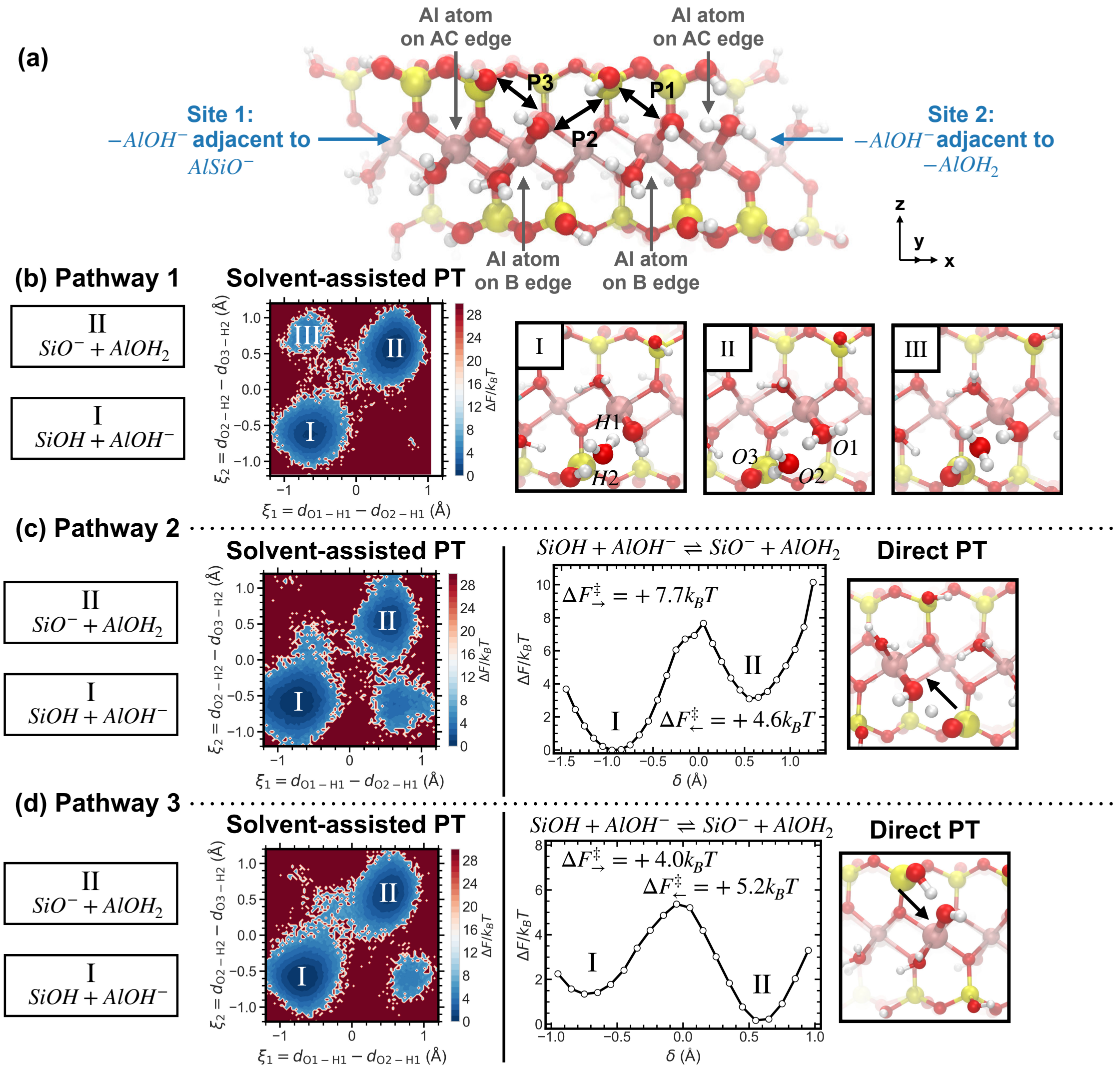

Figure S13: Proton transfer mechanisms at the B edge of montmorillonite under aqueous conditions. (a) Frontal view of the B edge. Black arrows indicate three distinct proton transfer pathways involving $-$SiOH and $-$AlOH$^-$ groups. (b) Pathway 1: Solvent-assisted proton transfer observed in neutral solution. Left: two-dimensional free energy surface projected onto collective variables $\xi_1 = d_{\mathrm{O1-H1}} - d_{\mathrm{O2-H1}}$ and $\xi_2 = d_{\mathrm{O2-H2}} - d_{\mathrm{O3-H2}}$, in units of Å. Right: representative snapshots illustrating key intermediates along the reaction coordinate. (c) Pathway 2: Proton transfer observed in basic solution. Left: free energy surface same as in (b), based on solvent-assisted dynamics. Right: free energy profile for the corresponding direct proton transfer pathway observed in neutral solution. (d) Pathway 3: Proton transfer observed in basic solution. Left: solvent-assisted free energy surface, as defined above. Right: free energy profile for the direct proton transfer pathway under basic conditions.

4.4 Proton Transfer Events at the AC Edge

AC edge sites also exhibit distinct reactivity patterns. Although relatively stable under acidic and neutral conditions, surface groups such as $-SiOH$ or $-AlOH_2$ can undergo deprotonation through reactions with hydroxide ions in basic solution, which in turn may initiate subsequent proton transfer cascades.

Two representative mechanisms are shown in Figure S14. The first is a direct PT reaction involving $-SiOH$ and $-AlOH^-$ groups, leading to the formation of $-SiO^-$ and $-AlOH_2$. While either configuration may serve as the initial state in simulations, the associated one-dimensional PTFEL was constructed along the forward reaction pathway, $-SiOH + AlOH^- \longrightarrow SiO^- + AlOH_2$, using a signed reaction coordinate δ. The resulting free energy profile shows a lower forward barrier ($\Delta F^{\ddagger}_{\rightarrow} = +2.2\,k_BT$) compared to the reverse ($\Delta F^{\ddagger}_{\leftarrow} = +3.8\,k_BT$), indicating a thermodynamic preference for the deprotonated $-SiO^-$ and protonated $-AlOH_2$ state.

The second mechanism involves a solvent-assisted PT between two $-SiOH$ groups, where one site was already deprotonated to form $-SiO^-$. The corresponding 2D PTFEL reveals that the transition between stable states I and II lacks continuous low-energy pathways, consistent with an isolated, irreversible transfer event rather than dynamic back-and-forth exchange. This interpretation is further supported by trajectory analysis, which shows that such transfers occurred only once or twice over the full 1.2 ns simulations, making it challenging to resolve a well-sampled free energy pathway for this mechanism. Interestingly, a third local minimum (state III) appears on the 2D landscape, corresponding to a stable but non-reactive solvation structure in which both hydrogen atoms of the bridging water molecule point toward surface groups. This orientation is not conducive to PT, highlighting the influence of local hydrogen-bonding patterns. These findings underscore that not all solvent-bridged geometries facilitate efficient PT: the directionality and strength of hydrogen bonds within the solvation network critically determine whether a bridging water molecule can support PT between surface groups.

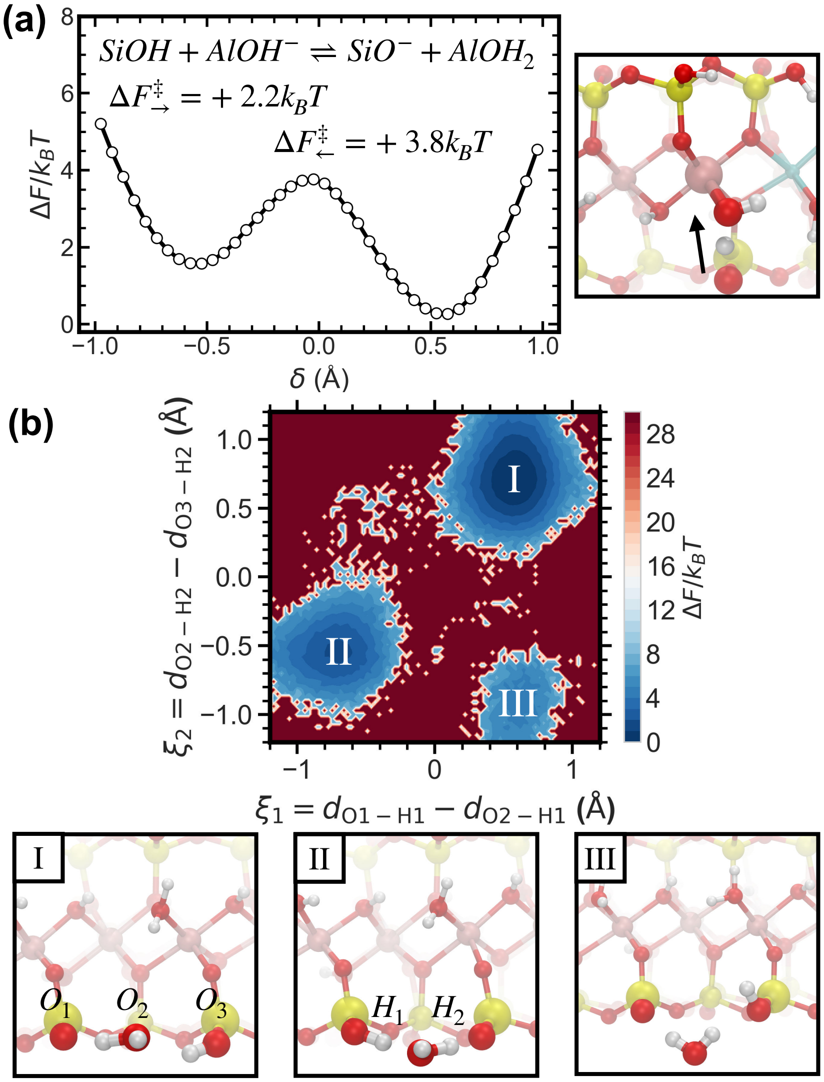

Figure S14: Proton transfer processes on the AC edge of the montmorillonite nanoparticle in basic solution. (a) Free energy profile for a direct proton transfer reaction on the AC edge, involving surface groups not adjacent to B edge junctions: $\mathrm{SiOH} + \mathrm{AlOH}^- \rightleftharpoons \mathrm{SiO}^- + \mathrm{AlOH}_2$. (b) Two-dimensional free energy surface for a solvent-assisted proton transfer process between $-\mathrm{SiO}^-$ and $-\mathrm{SiOH}$ groups, plotted as a function of ξ_1 and ξ_2 (in Å)

4.5 Water-mediated Multi-step Proton Transfer Events

In basic solution environments, we identified characteristic multi-step PT events at the AC edge, in which protons migrate between surface functional groups via bridging water molecules. As illustrated in Figure S15, two representative PT pathways were observed: (a) between adjacent −SiOH and $-SiO^-$ groups, and (b) between a −SiOH and a neighboring $-AlOH^-$ group. These PT events occur through water-mediated mechanisms involving transient configurations that resemble hydroxide-like species. The sequence of snapshots (Structures I–IV) clearly demonstrates that the proton is not transferred as a single, localized particle in a continuous fashion. Instead, the process proceeds via a delocalized proton hole propagating through the hydrogen-bond network, characteristic of a Grotthuss-like transfer mechanism. This pathway underscores the dynamic role of water in facilitating long-range PT by stabilizing transient intermediate states and reducing the energetic barriers associated with proton relocation.

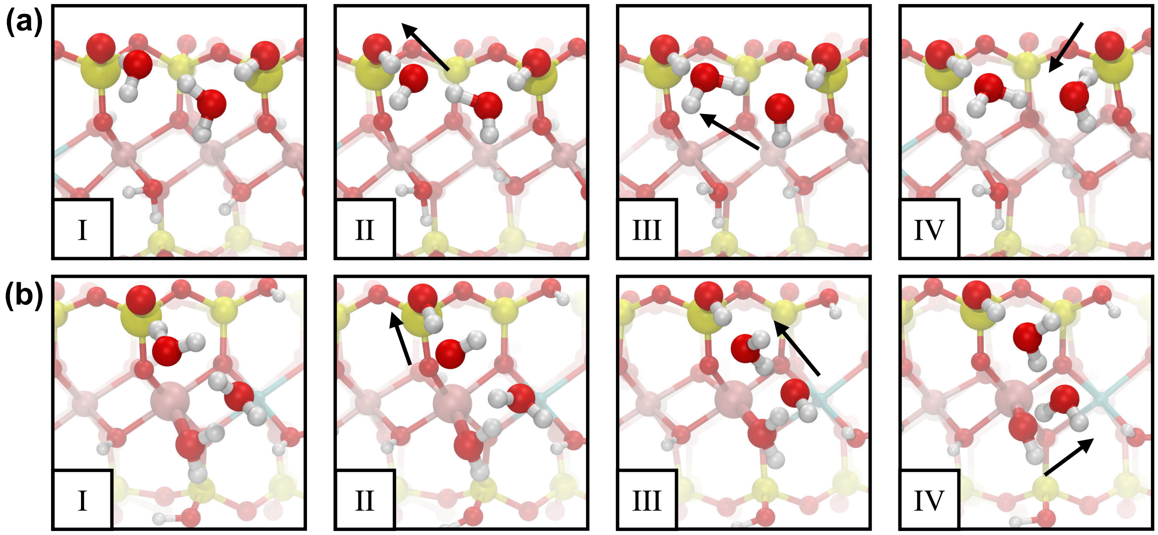

Figure S15: Representative snapshots illustrating water-mediated multi-step proton transfer events at the montmorillonite–water interface. (a) Proton migration between −SiOH and $-SiO^-$ groups. (b) Proton migration between −SiOH and $-AlOH^-$ groups. Each panel consists of four snapshots (Structures I–IV), which depict the sequential transfer of a proton through bridging water molecules. Arrows indicate the direction of proton transfer.

4.6 Isomorphic substitution influence in acidic and basic solution

In acidic conditions, isomorphic substitution significantly enhances the reactivity of otherwise inert edge motifs. As discussed above, the −AlAlOH group, typically unreactive, upon substitution to form −AlMgOH can be readily protonated by a hydronium ion from the bulk. Notably, in neutral solution, the −AlMgOH site was occasionally observed to transiently acquire a proton from a neighboring −SiOH group, although this proton was often released shortly thereafter. Similarly, both $-SiAlO^-$ and $-SiMgO^-$ groups are capable of undergoing protonation in acidic solution. The corresponding PTFELs for reactions involving hydronium ions are shown in Figure S16a. For $-SiAlO^-$, the free energy profile features two clear minima, corresponding to the protonated and deprotonated states. In contrast, the profile for $-SiMgO^-$ exhibits only a single minimum, representing the protonated −SiMgOH state. This indicates a strong thermodynamic preference for proton retention at the Mg-substituted site. The result is consistent with previously reported *ab initio* pK_a calculations, which place −SiMgOH at 4.2 and −SiAlOH at 1.7.[24]

Under basic conditions, isomorphic substitution similarly stabilizes protonated forms. As shown in Figure S16b, $-MgOH_2$ groups located on the AC edge (away from B-edge junctions) exhibit a pronounced asymmetry in their free energy profile: the barrier for deprotonation is substantially higher than that for reprotonation. This thermodynamic bias implies that even after donating a proton to a hydroxide ion, $-MgOH_2$ quickly regains a proton from neighboring −SiOH, maintaining its protonated state. This observation is also consistent with previous first-principles molecular dynamics studies, which report that Mg-substituted hydroxyl groups on montmorillonite edges exhibit significantly elevated pK_a values (up to 13.2), indicating a strong preference for remaining protonated under typical pH conditions.[23] Our simulations further reveal that despite this stability, $-MgOH_2$ groups can occasionally deprotonate and transiently participate in local proton transfer events under neutral conditions. Such behavior, not captured by static pK_a values, highlights their potential role in dynamic interfacial charge redistribution.

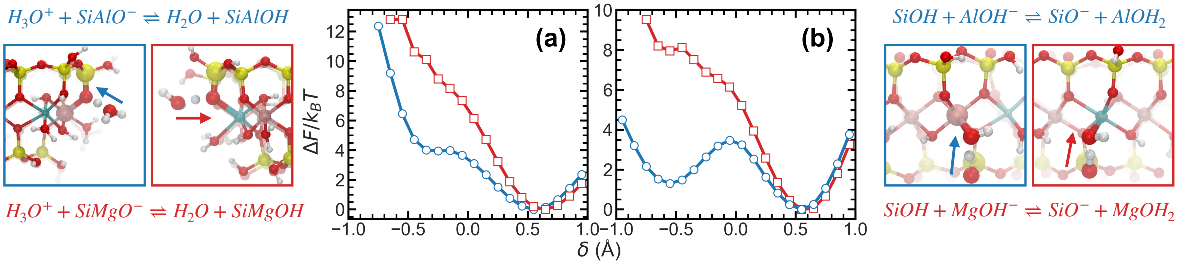

Figure S16: Effect of isomorphic substitution (Mg for Al) on the free energy profiles of direct proton transfer reactions in the montmorillonite nanoparticle system. Each panel compares two scenarios: red curves (highlighted by red frames) correspond to Mg-substituted sites, and blue curves (highlighted by blue frames) correspond to Al-only environments. For each case, the associated reaction equations and representative structures are shown alongside, with arrows indicating the direction of proton transfer. (a) Free energy profile for proton transfer between a hydronium ion (H_3O^+) and either $SiAlO^-$ or $SiMgO^-$ in acidic solution. (b) Free energy profile for a direct proton transfer on the AC edge, involving −SiOH and either $-AlOH^-$ or $-MgOH^-$ groups that are not located near B-edge junctions, in basic solution.

4.7 Proton Transfer Events of −AlMgOH

In the main text, we demonstrated that under acidic conditions, −AlMgOH groups located at the intersection of two AC edges can readily undergo protonation by hydronium ions, forming $-\text{AlMgOH}_2{}^+$. In addition to this process, further analysis of trajectories under neutral conditions revealed transient PT events between neighboring −SiOH and −AlMgOH groups. These interactions involve a spontaneous attempt by −AlMgOH to acquire a proton from the adjacent −SiOH, thereby forming a transiently protonated $-\text{AlMgOH}_2{}^+$ species. However, this protonation is short-lived, with the proton quickly returning to the original donor site within approximately 100 fs. To better understand the thermodynamic profile of this process, we computed the PTFEL for this direct PT. As shown in Figure S17, the protonation of −AlMgOH by −SiOH is associated with a significant free energy barrier ($\Delta F^{\ddagger}_{\rightarrow} = 7.5\, k_B T$), whereas the reverse reaction has a much lower barrier ($\Delta F^{\ddagger}_{\leftarrow} = 0.5\, k_B T$). These results suggest that while −AlMgOH groups are capable of temporarily accepting a proton in neutral environments, the process is thermodynamically unfavorable and kinetically unstable, resulting in rapid reversion to the deprotonated state.

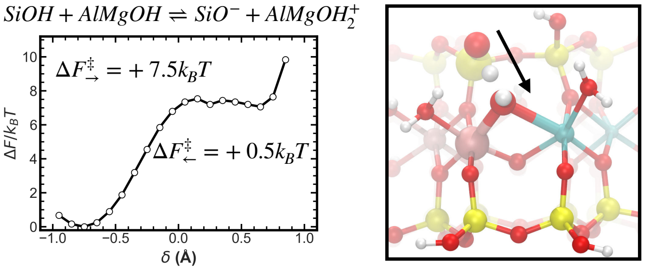

Figure S17: Free energy profile of a proton transfer reaction between a surface −SiOH group and a neighboring −AlMgOH site. The representative structure is shown adjacent to the energy curve, with an arrow indicating the direction of proton transfer.

References

[1] Necip Güven. The crystal structures of 2 m 1 phengite and 2 m 1 muscovite. *Zeitschrift für Kristallographie - Crystalline Materials*, 134:196–212, 1971.

[2] G. Norman White and L. W. Zelazny. Analysis and implications of the edge structure of dioctahedral phyllosilicates. *Clays and Clay Minerals*, 36(2):141–146, 1988.

[3] Sergey V. Kraevsky, Christophe Tournassat, Marylène Vayer, Fabienne Warmont, Sylvain Grangeon, Brice F. Ngouana Wakou, and Andrey G. Kalinichev. Identification of montmorillonite particle edge orientations by atomic-force microscopy. *Applied Clay Science*, 186:105442, 2020.

[4] Aric G. Newton and Garrison Sposito. Molecular dynamics simulations of pyrophyllite edge surfaces: Structure, surface energies, and solvent accessibility. *Clays and Clay Minerals*, 63(4):277–289, 2015.

[5] Aric G. Newton, Kideok D. Kwon, and Dae-Kyo Cheong. Edge structure of montmorillonite from atomistic simulations. *Minerals*, 6(2), 2016.

[6] Kideok D. Kwon and Aric G. Newton. Structure and stability of pyrophyllite edge surfaces: Effect of temperature and water chemical potential. *Geochimica et Cosmochimica Acta*, 190:100–114, 2016.

[7] Aidan P. Thompson, H. Metin Aktulga, Richard Berger, Dan S. Bolintineanu, W. Michael Brown, Paul S. Crozier, Pieter J. in 't Veld, Axel Kohlmeyer, Stan G. Moore, Trung Dac Nguyen, Ray Shan, Mark J. Stevens, Julien Tranchida, Christian Trott, and Steven J. Plimpton. Lammps - a flexible simulation tool for particle-based materials modeling at the atomic, meso, and continuum scales. *Computer Physics Communications*, 271:108171, 2022.

[8] Ilyes Batatia, Philipp Benner, Yuan Chiang, Alin M. Elena, Dávid P. Kovács, Janosh Riebesell, Xavier R. Advincula, Mark Asta, Matthew Avaylon, William J. Baldwin, Fabian Berger, Noam Bernstein, Arghya Bhowmik, Samuel M. Blau, Vlad Cărare, James P. Darby, Sandip De, Flaviano Della Pia, Volker L. Deringer, Rokas Elijošius, Zakariya El-Machachi, Fabio Falcioni, Edvin Fako, Andrea C. Ferrari, Annalena Genreith-Schriever, Janine George, Rhys E. A. Goodall, Clare P. Grey, Petr Grigorev, Shuang Han, Will Handley, Hendrik H. Heenen, Kersti Hermansson, Christian Holm, Jad Jaafar, Stephan Hofmann, Konstantin S. Jakob, Hyunwook Jung, Venkat Kapil, Aaron D. Kaplan, Nima Karimitari, James R. Kermode, Namu Kroupa, Jolla Kullgren, Matthew C. Kuner, Domantas Kuryla, Guoda Liepuoniute, Johannes T. Margraf, Ioan-Bogdan Magdău, Angelos Michaelides, J. Harry Moore, Aakash A. Naik, Samuel P. Niblett, Sam Walton Norwood, Niamh O'Neill, Christoph Ortner, Kristin A. Persson, Karsten Reuter, Andrew S. Rosen, Lars L. Schaaf, Christoph Schran, Benjamin X. Shi, Eric Sivonxay, Tamás K. Stenczel, Viktor Svahn, Christopher Sutton, Thomas D. Swinburne, Jules Tilly, Cas van der Oord, Eszter Varga-Umbrich, Tejs Vegge, Martin Vondrák, Yangshuai Wang, William C. Witt, Fabian Zills, and Gábor Csányi. A foundation model for atomistic materials chemistry, 2024.

[9] Thomas D. Kühne, Marcella Iannuzzi, Mauro Del Ben, Vladimir V. Rybkin, Patrick Seewald, Frederick Stein, Teodoro Laino, Rustam Z. Khaliullin, Ole Schütt, Florian Schiffmann, Dorothea Golze, Jan Wilhelm, Sergey Chulkov, Mohammad Hossein Bani-Hashemian, Valéry Weber, Urban Borštnik, Mathieu Taillefumier, Alice Shoshana Jakobovits, Alfio Lazzaro, Hans Pabst, Tiziano

Müller, Robert Schade, Manuel Guidon, Samuel Andermatt, Nico Holmberg, Gregory K. Schenter, Anna Hehn, Augustin Bussy, Fabian Belleflamme, Gloria Tabacchi, Andreas Glöß, Michael Lass, Iain Bethune, Christopher J. Mundy, Christian Plessl, Matt Watkins, Joost VandeVondele, Matthias Krack, and Jürg Hutter. Cp2k: An electronic structure and molecular dynamics software package - quickstep: Efficient and accurate electronic structure calculations. *The Journal of Chemical Physics*, 152(19):194103, 05 2020.

[10] Niamh O'Neill, Benjamin X. Shi, Kara Fong, Angelos Michaelides, and Christoph Schran. To pair or not to pair? machine-learned explicitly-correlated electronic structure for nacl in water. *The Journal of Physical Chemistry Letters*, 15(23):6081–6091, 2024.

[11] John P. Perdew, Kieron Burke, and Matthias Ernzerhof. Generalized gradient approximation made simple. *Phys. Rev. Lett.*, 77:3865–3868, Oct 1996.

[12] Yingkai Zhang and Weitao Yang. Comment on "generalized gradient approximation made simple". *Phys. Rev. Lett.*, 80:890–890, Jan 1998.

[13] Stefan Grimme, Jens Antony, Stephan Ehrlich, and Helge Krieg. A consistent and accurate ab initio parametrization of density functional dispersion correction (dft-d) for the 94 elements h-pu. *The Journal of Chemical Physics*, 132(15):154104, 04 2010.

[14] Ondrej Marsalek and Thomas E. Markland. Quantum dynamics and spectroscopy of ab initio liquid water: The interplay of nuclear and electronic quantum effects. *The Journal of Physical Chemistry Letters*, 8(7):1545–1551, 2017.

[15] Sam Shepherd, Gareth A. Tribello, and David M. Wilkins. A fully quantum-mechanical treatment for kaolinite. *The Journal of Chemical Physics*, 158(20):204704, 05 2023.

[16] Randall T. Cygan, Jian Jie Liang, and Andrey G. Kalinichev. Molecular models of hydroxide, oxyhydroxide, and clay phases and the development of a general force field. *Journal of Physical Chemistry B*, 108:1255–1266, 2004.

[17] S. Goedecker, M. Teter, and J. Hutter. Separable dual-space gaussian pseudopotentials. *Phys. Rev. B*, 54:1703–1710, Jul 1996.

[18] Ilyes Batatia, Dávid Péter Kovács, Gregor N. C. Simm, Christoph Ortner, and Gábor Csányi. Mace: Higher order equivariant message passing neural networks for fast and accurate force fields, 2023.

[19] Dávid Péter Kovács, Ilyes Batatia, Eszter Sára Arany, and Gábor Csányi. Evaluation of the mace force field architecture: From medicinal chemistry to materials science. *The Journal of Chemical Physics*, 159(4):044118, 07 2023.

[20] Jung Hoo Lee and Stephen Guggenheim. Single crystal x-ray refinement of pyrophyllite-1tc. *American Mineralogist*, 66(3-4):350–357, 04 1981.

[21] Lawrie B. Skinner, Congcong Huang, Daniel Schlesinger, Lars G. M. Pettersson, Anders Nilsson, and Chris J. Benmore. Benchmark oxygen-oxygen pair-distribution function of ambient water from x-ray diffraction measurements with a wide q-range. *The Journal of Chemical Physics*, 138(7):074506, 02 2013.

[22] Xavier R. Advincula, Kara D. Fong, Angelos Michaelides, and Christoph Schran. Protons accumulate at the graphene–water interface. *ACS Nano*, 19(18):17728–17737, 2025.

[23] Xiandong Liu, Xiancai Lu, Michiel Sprik, Jun Cheng, Evert Jan Meijer, and Rucheng Wang. Acidity of edge surface sites of montmorillonite and kaolinite. *Geochimica et Cosmochimica Acta*, 117:180–190, 2013.

[24] Xiandong Liu, Jun Cheng, Michiel Sprik, Xiancai Lu, and Rucheng Wang. Surface acidity of 2:1-type dioctahedral clay minerals from first principles molecular dynamics simulations. *Geochimica et Cosmochimica Acta*, 140:410–417, 2014.

[25] Johannes Kästner and Walter Thiel. Bridging the gap between thermodynamic integration and umbrella sampling provides a novel analysis method: "umbrella integration". *The Journal of Chemical Physics*, 123(14):144104, 10 2005.

[26] Ivaylo Ivanov, Bin Chen, Simone Raugei, and Michael L. Klein. Relative pka values from first-principles molecular dynamics: the case of histidine deprotonation. *The Journal of Physical Chemistry B*, 110(12):6365–6371, 2006.

[27] Ruiyu Wang, Vincenzo Carnevale, Michael L. Klein, and Eric Borguet. First-principles calculation of water pka using the newly developed scan functional. *The Journal of Physical Chemistry Letters*, 11(1):54–59, 2020.

[28] Yair Litman and Angelos Michaelides. Entropy governs the structure and reactivity of water dissociation under electric fields. *Journal of the American Chemical Society*, 147(49):44885–44894, 2025.

[29] Xavier R. Advincula, Yair Litman, Kara D. Fong, William C. Witt, Christoph Schran, and Angelos Michaelides. How reactive is water at the nanoscale and how to control it? 2025.

[30] Masakazu Matsumoto. Relevance of hydrogen bond definitions in liquid water. *The Journal of Chemical Physics*, 126(5):054503, 02 2007.
