# Supplementary material for: Clay Edges Are Dynamic Proton-Conducting Networks Modulated by Structure and pH
Source: J Phys Chem Lett. 2026 Feb 23;17(9):2679–88. doi: 10.1021/acs.jpclett.5c03748 (PMC12969369; doi:10.1021/acs.jpclett.5c03748)
Supplement: Supplementary file 2 [file jz5c03748_si_002.pdf]

jz-2025-03748z.R1

Name: Peer Review Information for "Clay Edges Are Dynamic Proton-conducting Networks Modulated by Structure and pH"

First Round of Reviewer Comments

Reviewer: 1

Comments to the Author

The authors apply machine learning potentials to study proton transfer dynamics and mechanisms on X-OH groups at montmorillonite edge surfaces. The paper is well written and researched. As written, it is interesting to a rather specialized audience. It may be of wider interest to a general audience with more work. Several aspects of the model can also use clarification. I recommend a significant revision.

1. The main concern with this work is the disconnect between motivation and results. The highlighted results, focusing on fine details of interfacial proton transfer mechanisms, free energies barriers, etc., do not obviously translate into relevant science questions and applications discussed in the Introduction (e.g., surface reactivity); the authors may not have focused on presenting the most interesting results to a wide audience.

The most relevant result might be Fig. 2c, but it is highly compressed. Please expand on this.

2. Do the ML potentials yield accurate pKa for these in "static" groups, calculated by AIMD simulations? This seems an obvious check of the MLFF. To my knowledge, this has not been conducted (I searched for 'pK').

3. "Acidic" and "basic" condition wordings are used throughout the paper and the SI. Only one sentence indicates what these are (line 244, pH=0.44 and 13.56). Please change all wordings to pH=0.4 and pH=13.6 throughout for clarity. Are these pH values realistic/relevant for applications?

Do the clay crystal structure survive at these pH's?

4. How do the nanocrystal sizes associated with the models compare with experiments?

5. The relaxation time scales (e.g., Fig.2a) might be of interest to the broader computational community when modeling interfaces. However, the MLFF seems to have a range of 5 Angstrom (SI Sec. 1.4). Do the H+/OH- kinetics reflect long range electrostatic interactions between the H+/OH- and the surfaces, or do they merely reflect diffusion (and therefore overestimate the relaxation time scale)?

6. Please list the AIMD trajectory lengths associated with Table S2.

7. The D3 correction to DFT is found to be problematic for ions in water [J. Phys. Chem. Lett. 2023, 14, 4403. Please comment on its suitability for oxide surfaces.

Reviewer: 2

Comments to the Author

I have read the the article, "Clay Edges Are Dynamic Proton-conducting Networks

Modulated by Structure and pH" with great interest. This is a nice study with potentially interesting findings relevant to the geochemical importantly the colloidal community.

My first impression is that this too long and too comprehensive for a JPC-letter. This is a an issue for the editor to resolve. I have made a note to him.

Nevertheless it is a nice study, but no simple message that is required for letters (see guidelines) is conveyed.

A letter style result would be to say something definitive regarding the pKa (under solution conditions) for these important systems regarding curvature, size, and even the loading of the defects. This would have profound implications for colloidal studies of different size nanoparticle and the plethora of phenomena related to charge-charge attractions and surface charge regulation as they stated early in the manuscript.

What is presented is a very high quality and detailed study of small (very small) nanoparticles under "pH."   First, I think the authors should embrace a "notional" scale rather than an absolute values they state. You can't determine the system pH without rigorous analyses of simulations in conjunction with non-ideal solution thermodynamics and addressing how the activity of H3O+ is related to the concentration of H3O+. In fact, if the authors state they can do this, this would be a topic worthy of a letter contribution. The setup appears to be composition-defined (fixed numbers of H_3O^+/Cl^- or OH^-/Na^+ added initially). Because the clay edge buffers protons, the effective acidity can drift during the trajectory, consistent with their tracking of net proton excess ($\#H_3O^+ - \#OH^-$).

What I liked about the manuscript:

1. The isomorphic substitution (Al/Mg) results are fascinating.   This is a highlight of the study. One could write a short letter about this phenomena.

2. The finding of less proton hopping events in acidic conditions, although a very interesting and solid finding, seems rather intuitive. Again, I think have a molecular verification of intuition via simulation is an important self-consistent check, it doesn't strike me as s a "letter" quality result.

3. The resulting rich proton dynamics on edges vs surfaces is also very interesting.

Some ideas for improvement:

1. As stated above, the manuscript would benefit from explicitly defining pH and from reporting how much this metric changes over time (and over the analysis window). But, there is value to saying that the study takes place on a "notional" scale of "low" , "medium" (near neutral) and "high" pH environments and would relabel their figures.

2. t should be feasible to analyze a representative subset of the existing trajectories to quantify surface charge-density changes for the studied systems. This would be of broad interest to the geochemistry community.

3. How sensitive are the reported conclusions to the choice of ML potential architecture and training framework? In particular, do the free energies remain consistent when using an alternative ML engine/potential (trained to comparable reference data and accuracy) rather than MACE?

In summary the work is of high quality and I would accept this immediately with the suggested improvements if it were a standard JPC contribution. It's an important study by a fantastic group. My complaints are the JPC-letter format. It seems forced. This figures are nice but huge and complex. It's a detailed read with lots of rich and important details. Again, my only complaint is that it is too much for a latter journal.

If the other referees and the Editor agree this is a proper letter I will go along.

It is an important and well executed study that the practitioners in the field will enjoy and cite.

Author's Response to Peer Review Comments:

Referee response letter: Clay Edges Are Dynamic Proton-conducting Networks Modulated by Structure and pH

We repeat all reports in full length *in italics*, respond in a point-by-point manner to all questions and suggestions. We show in red added or changed text in the manuscript.

R1. RESPONSE TO REFEREE 1

Referee's Summary

> *Recommendation: This paper may be publishable, but major revision is needed.*
>
> *Comments: The authors apply machine learning potentials to study proton transfer dynamics and mechanisms on X-OH groups at montmorillonite edge surfaces. The paper is well written and researched. As written, it is interesting to a rather specialized audience. It may be of wider interest to a general audience with more work. Several aspects of the model can also use clarification. I recommend a significant revision.*

Response to Summary

We sincerely thank the reviewer for their careful reading of the manuscript and for their thoughtful and constructive feedback. All questions and comments have been addressed in the revised manuscript, as detailed below. We believe that the resulting revisions have strengthened the clarity, scope, and overall impact of the Letter.

Comment 1.1.

> *The main concern with this work is the disconnect between motivation and results. The highlighted results, focusing on fine details of interfacial proton transfer mechanisms, free energies barriers, etc., do not obviously translate into relevant science questions and applications discussed in the Introduction (e.g., surface reactivity); the authors may not have focused on presenting the most interesting results to a wide audience.*
>
> *The most relevant result might be Fig. 2c, but it is highly compressed. Please expand on this.*

Response to Comment 1.1.

We thank the reviewer for this important and constructive comment. We agree that, in the original version, the breadth and level of mechanistic detail may have obscured the central message and weakened the connection between the motivating questions outlined in the Introduction and the Results presented.

In the revised manuscript, we have refocused the narrative around a single overarching finding: that montmorillonite edge surfaces behave as amphoteric, dynamically adaptive proton-exchange networks whose reactive state is modulated by local chemical environment. Detailed analyses of proton transfer pathways and free energy profiles are now positioned as supporting evidence rather than as primary results.

To strengthen the link to surface reactivity, we have revised Fig. 2 to explicitly include the time-dependent evolution of the nanoparticle surface charge (Fig. 2b). Together with the aqueous proton excess (Fig. 2a), this provides a direct and intuitive representation of surface charge regulation and buffering, which are central to the geochemical and colloidal processes discussed in the Introduction.

Regarding Fig. 2c, we clarify that this panel is intended to illustrate representative population shifts of surface functional groups, while the identification of reactive sites and their pH-dependent behavior is based on trajectory analysis across all nanoparticle configurations. In the revised text, we explicitly emphasize the general patterns of surface reactivity that emerge across systems, and we use Fig. 2c as a compact visualization of these shared trends rather than an exhaustive catalog of site-specific behavior.

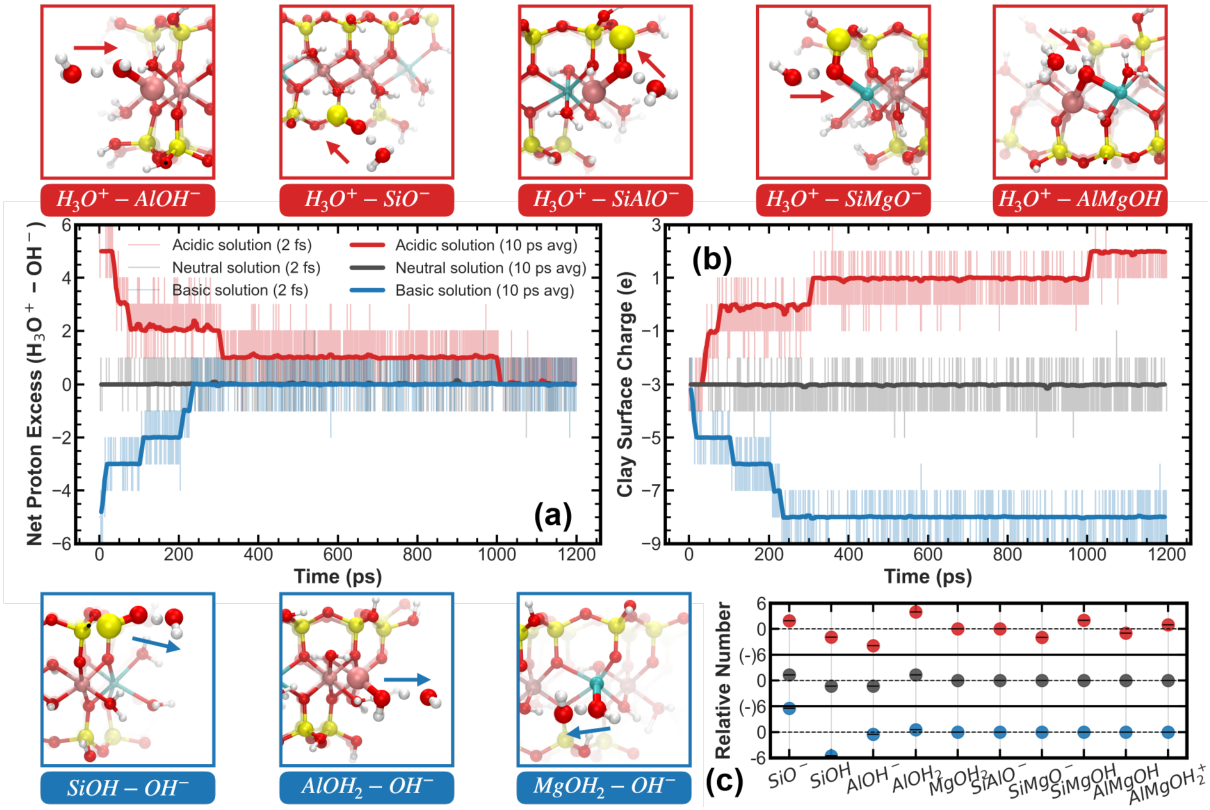

Figure R1: Protonation and deprotonation behavior of the montmorillonite nanoparticle in different aqueous environments. (a) Time evolution of the net proton excess in the aqueous phase (defined as the number of hydronium ions minus hydroxide ions) over 1200 ps. (b) Time evolution of the net surface charge of the montmorillonite nanoparticle, derived from the intrinsic negative structural charge of the clay due to isomorphic substitution, together with protonation and deprotonation of the nanoparticle through proton exchange with the aqueous solution. Transparent curves represent data sampled every 2 fs; solid lines denote a 10 ps average. (c) Relative abundance of various surface functional groups during the final 200 ps, compared to their initial populations; error bars are included but smaller than the symbol size. In all panels, colors denote the solution pH: red for acidic, black for neutral, and blue for basic conditions. Top panel: Surface groups that gain protons from hydronium ions under acidic conditions. Bottom panel: Surface groups that donate protons to hydroxide ions under basic conditions. Arrows indicate the direction of proton transfer, with representative surface reactions labeled below each group pair. Results shown correspond to one representative montmorillonite nanoparticle; analogous behavior for the other two configurations is provided in the Supplementary Information.

Finally, in response to the reviewer's comments regarding scope and clarity, we have reduced the overall length of the manuscript and streamlined the discussion. The revised version focuses more sharply on the central physical picture, with secondary analyses moved to the Supplementary Information where appropriate. This restructuring better aligns the manuscript with the *The Journal of Physical Chemistry Letters* format and makes the main conclusions more accessible to a broad readership.

Comment 1.2.

Do the ML potentials yield accurate pKa for these in ”static” groups, calculated by AIMD simulations? This seems an obvious check of the MLFF. To my knowledge, this has not been conducted (I searched for ’pKa’).

Response to Comment 1.2.

We thank the reviewer for this important comment. We fully agree that acid–base properties, and in particular pK_a values, provide a stringent and meaningful test of machine-learning force fields.

To address this point, we examined whether pK_a-related information can be inferred from our neutral-water simulations using population statistics of protonated and deprotonated surface states. In principle, such an analysis can be formulated within a Henderson–Hasselbalch framework under conditions of known pH. However, under neutral conditions (pH ≈ 7), many surface sites remain effectively permanently protonated over the accessible simulation timescales, which precludes the extraction of meaningful numerical pK_a values and allows only lower bounds to be inferred. To account for this limitation, we adopted a conservative criterion and classified a site as “active” only if it exhibits a deprotonated population exceeding 0.01, corresponding to an effective pK_a ≈ 9 at pH ≈ 7.

Rather than attempting a direct numerical comparison between population-derived pK_a values and intrinsic pK_a values reported in the literature, we therefore assessed the consistency between relative proton activity inferred from our simulations and reported pK_a trends. This analysis has now been explicitly added to the Supplementary Section S2.5:

” Acid–base properties of surface functional groups, and in particular their intrinsic pK_a values, provide a stringent benchmark for atomistic models of reactive mineral–water interfaces. Here, we examine whether relative trends in surface proton activity inferred from our neutral-water simulations are consistent with pK_a values reported in prior *ab initio* studies.

In principle, under conditions of known pH (here nominally pH ≈ 7), a Henderson–Hasselbalch–type relation may be used to estimate pK_a values from the relative populations of protonated and deprotonated states,

$$\mathrm{p}K_a = \mathrm{pH} - \log_{10}\left(\frac{P_{\mathrm{deprot}}}{P_{\mathrm{prot}}}\right); \qquad \text{(R1)}$$

where P_{prot} and P_{deprot} denote the fraction of time a given surface oxygen is found in protonated and deprotonated states, respectively.

However, an important methodological limitation arises in the context of neutral-water simulations. At pH ≈ 7, many surface sites remain effectively permanently protonated over

the accessible simulation timescales. For such sites, population ratios do not permit the extraction of a meaningful numerical pK_a value; at best, lower bounds can be inferred. To make this distinction explicit, we therefore introduce a conservative criterion and classify a site as "active" only if its deprotonated population exceeds 0.01, corresponding to an effective pK_a ≈ 9 at pH ≈ 7.

Using this criterion, we analyzed the fraction of active sites for different surface functional groups across the three nanoparticle models. Averaged over all systems, the fraction of active sites follows the ordering

$$\text{B-edge Al(OH)}_2 > \text{AC-edge Al(OH)}_2 > \text{SiOH} > \text{Mg(OH)}_2; \quad \text{(R2)}$$

with approximate active fractions of 69%, 23%, 10%, and 0%, respectively.

Importantly, this ordering is fully consistent with intrinsic pK_a values reported in previous AIMD studies

(pK_a ≈ 3:1 for B-edge $Al(OH)_2$, 5.5 for AC-edge $Al(OH)_2$, ~7.0 for SiOH, and ~13.2 for $Mg(OH)_2$).[1,2] Although absolute pK_a values cannot be directly extracted from neutral-water simulations, the relative proton activity of different surface groups inferred here is therefore in qualitative agreement with established firstprinciples results. "

Regarding validation of the machine-learning force field, we emphasize that acid–base equilibria are fundamentally governed by free-energy differences between protonated and deprotonated states, which in turn depend on an accurate description of proton-transfer energetics. In this work, rather than attempting a direct numerical comparison of population-derived pK_a values from neutral simulations with intrinsic pK_a values obtained from isolated-site AIMD calculations, we validated the ML potential by comparing proton-transfer free-energy profiles obtained with the MLFF against reference AIMD simulations under identical conditions. As shown in Fig. R2, the ML potential accurately reproduces the AIMD free-energy profiles for proton transfer in bulk water for both excess-proton and proton-deficient defects, capturing barrier heights, curvatures, and relative state stabilities along the reaction coordinate.

In this sense, agreement in proton-transfer free energies provides a stringent and physically meaningful valida-

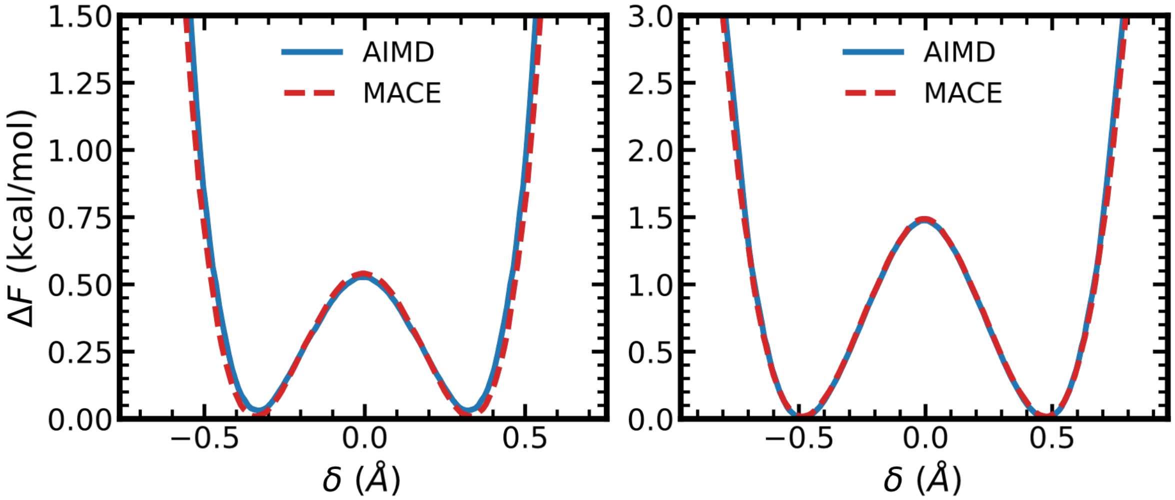

Figure R2: Proton-transfer free energy profiles for aqueous protonic defects in bulk solution. Left: proton transfer between a hydronium ion and a neighboring water molecule. Right: proton transfer between a hydroxide ion and a neighboring water molecule. In both cases, the AIMD reference free-energy curve is shown as a blue solid line,[3,4] while the MACE prediction is shown as a red dashed line.

tion of the ML potential for modeling proton affinity and proton exchange processes in aqueous environments. We therefore believe that, in the present context, validation against AIMD proton-transfer free energies constitutes a more appropriate and robust test of the MLFF than a direct numerical comparison of population-based pK_a values from neutral simulations with intrinsic pK_a values reported in the literature.

Finally, we note that the Methods section of the main text has also been updated to explicitly emphasize the scope of the MLFF validation.

" Further validation confirmed the model's reliability in reproducing interfacial energetics, clay lattice structures, bulk water properties and proton transfer free energies (see Supplementary Section S2). "

Comment 1.3.

"Acidic" and "basic" condition wordings are used throughout the paper and the SI. Only one sentence indicates what these are (line 244, pH=0.44 and 13.56). Please change all wordings to pH=0.4 and pH=13.6 throughout for clarity. Are these pH values realistic/relevant for applications?

Do the clay crystal structure survive at these pH's?

Response to Comment 1.3.

We thank the reviewer for raising this important point. We agree that the use of the terms "acidic" and "basic" may be misleading if interpreted as well-defined, externally maintained macroscopic pH conditions.

In the simulations, the acidity is defined by the initial composition of the aqueous phase, namely by adding a fixed number of hydronium/chloride or hydroxide/sodium ions to the simulation box. The quoted values (pH ≈ 0.44 and 13.56) correspond to the equivalent bulk pH of this initial composition and are provided only to give an intuitive reference point. During the simulations, no additional ions are supplied to maintain a constant pH, and proton exchange with the clay nanoparticle leads to a time-dependent drift in effective acidity. We have modified the relevant text to be more specific:

" To examine the pH-dependent reactivity of edge sites, each nanoparticle was simulated in aqueous environments prepared at initial acidic (pH 0.44), neutral (pH 7), and basic (pH 13.56) conditions (Figures 1d–f), including respective counter ions to retain charge neutrality. To examine the pH-dependent reactivity of edge sites, each nanoparticle was simulated in aqueous environments prepared at initial acidic (pH 0.44), neutral (pH 7), and basic (pH 13.56) conditions (Figures 1d–f), including respective counter ions to retain charge neutrality. These pH values represent the equivalent bulk pH associated with the initial concentrations of hydronium or hydroxide ions in the simulation cell and are provided as a reference for comparison. During the simulations, no external buffering or ion exchange was imposed to maintain a fixed pH; instead, proton exchange between the aqueous phase and the clay edge surfaces was allowed to proceed freely, leading to time-dependent changes in the effective acidity of the solution. Accordingly, the terms acidic, neutral, and basic environments are used throughout to denote these composition-defined conditions rather than strictly controlled macroscopic pH values. "

Because the system size is necessarily small, only a few excess protons or hydroxide ions are sufficient to correspond to extreme bulk pH values, while their absolute number remains limited relative to the number of reactive surface sites. Under these conditions, the simulations probe the response of the nanoparticle to acidic or basic environments rather than stable macroscopic pH conditions. We have therefore revised the manuscript to reduce emphasis on absolute pH values and instead consistently refer to acidic, neutral, and basic environments, with explicit clarification of how these are defined.

Regarding structural stability, we note that the simulations are not intended to represent long-term exposure of montmorillonite to strongly acidic or alkaline solutions under buffered conditions, where dissolution would be expected. Rather, they capture the short-time-scale proton exchange and surface reactivity of intact nanoparticles under different proton chemical potentials.

Comment 1.4.

How do the nanocrystal sizes associated with the models compare with experiments?

Response to Comment 1.4.

We thank the reviewer for raising this important point regarding the size of the modeled nanocrystals. Montmorillonite particles are intrinsically layered and plate-like. In terms of thickness, our models correspond to single-layer platelets, which is consistent with experimentally observed exfoliated or delaminated montmorillonite particles commonly encountered in aqueous environments.

In the lateral directions, the modeled nanoparticles have radii on the order of 1 nm, which is indeed smaller than typical experimental montmorillonite platelets, whose lateral dimensions are often irregular and span from tens of nanometers to the micrometer scale.[5,6] We acknowledge this difference. However, our primary focus is on the chemistry and dynamics of edge surfaces, which are local in nature and governed by the atomic-scale structure and composition of edge functional groups rather than by the overall lateral size of the particle.

From a computational perspective, it is worth noting that a number of previous force-field–based molecular dynamics and ab initio molecular dynamics studies have adopted clay nanoparticles of comparable lateral dimensions to investigate a range of physicochemical properties, including dehydration behavior in suspensions, aggregation mechanisms, and solid–liquid interfacial interactions.[7–11] In these studies, the lateral dimensions of the modeled nanoparticles typically range from a few nanometers up to approximately 10 nm.

In this context, our choice of laterally minimal but structurally representative nanoparticles reflects a balance between computational feasibility and chemical resolution. This allows us to resolve edge-specific proton transfer and surface reactivity at near first-principles accuracy, with mechanisms that are expected to be transferable to larger clay platelets.

To clarify this point in the manuscript, we have added the following sentence:

“ The atomic structures of the B and AC edge terminations are shown in Figures 1b and 1c, using minimal
yet structurally representative models commonly employed in atomistic simulations of clay nanoparticles.[7–11]
”

Comment 1.5.

The relaxation time scales (e.g., Fig.2a) might be of interest to the broader computational community when modeling interfaces. However, the MLFF seems to have a range of 5 Angstrom (SI Sec. 1.4). Do the H^+/OH^- kinetics reflect long range electrostatic interactions between the H^+/OH^- and the surfaces, or do they merely reflect diffusion (and therefore overestimate the relaxation time scale)?

Response to Comment 1.5.

We thank the reviewer for this insightful question. While the MACE model employs a radial cutoff of 5 ˚A for local interactions, this cutoff does not directly correspond to the physical interaction range sensed by reactive species in the simulation.

As described in the Methods and now clarified in the Supplementary Section 1.4, the model uses multiple message-passing layers, such that the effective receptive field is given by the product of the cutoff distance and the number of layers, reaching approximately 10 ˚A in the present configuration. This allows the model to capture short- and medium-range interactions extending beyond the nominal cutoff.

Specifically, we have revised the Methods section and expanded the Supplementary as follows:

" For this study, the MACE model was configured with two message-passing layers and four-body equivariant features, using a radial cutoff of 5 ˚A. explicit long-range interactions are not directly included, the receptive field of the model—defined by the product of the cutoff and the number of layers—effectively reaches 10 ˚A, which is sufficient to capture the relevant short- and medium-range interactions and to describe the dominant mechanisms controlling proton exchange and relaxation at the clay–water interface on the time scales investigated here. (see Methods)" and

" In this work, the MACE model was configured with two message-passing layers and four-body equivariant features, using a cutoff radius of 5 ˚A. explicit long-range electrostatics are not included, the effective receptive field of the model is given by the product of the cutoff distance and the number of message-passing layers, reaching approximately 10 ˚A. This interaction range is sufficient to capture the dominant short- and mediumrange interactions between solvated protons/hydroxide ions and clay edge surfaces in the present simulation cell. (see Supplementary Section 1.4) "

In the present simulation cell (approximately 36 × 36 × 22 ˚A^3), this receptive field spans a substantial fraction of the aqueous region and exceeds half of the nanoparticle diameter (16 ˚A). Consequently, hydronium and hydroxide ions remain within the effective sensing

range of the clay edge surfaces for much of the simulation, and their dynamics are not governed solely by bulk diffusion.

Accordingly, the observed relaxation time scales reflect a combination of diffusion and surface-mediated proton exchange processes rather than purely diffusion-limited behavior. While explicit long-range electrostatics are not included, the interaction range captured by the MACE model is sufficient to describe the dominant mechanisms controlling proton exchange and relaxation at the clay–water interface on the time scales investigated here.

Comment 1.6.

> *Please list the AIMD trajectory lengths associated with Table S2.*

Response to Comment 1.6.

We thank the reviewer for this comment. We have now explicitly added the AIMD trajectory lengths associated with Table S2 to the Supplementary Information. The revised text reads:

" The simulations were conducted at temperatures ranging from 298 K to 400 K with a time step of 0.5 fs, generating a total trajectory length of 50 ps for each condition listed in Table S2."

Comment 1.7.

> *The D3 correction to DFT is found to be problematic for ions in water [J. Phys. Chem. Lett. 2023, 14, 4403. Please comment on its suitability for oxide surfaces.*

Response to Comment 1.7.

We thank the reviewer for raising this important point. We are indeed aware of the documented limitations of the D3 dispersion correction for hydrated cations in bulk water, which originate from the use of neutral-atom reference dispersion coefficients and the lack of explicit treatment of changes in polarizability upon ionization.

With respect to oxide and clay mineral surfaces, we note that the physical context differs significantly from that of isolated ion hydration in bulk aqueous solution. At hydrated oxide or montmorillonite edge surfaces, the dominant interactions governing structure and reactivity are electrostatics, local coordination, and hydrogenbond networks involving surface hydroxyl groups and interfacial water, rather than dispersion-dominated ion–water

interactions. As a result, the known deficiencies of D3 for free hydrated cations are expected to play a much smaller role in determining the relative energetics and mechanisms relevant to surface protonation and proton transfer.

In the present work, revPBE-D3 is therefore not employed to claim quantitative accuracy for ion hydration or ion adsorption free energies. Instead, it is used as a consistent reference level for generating training data for the machine-learning potential, with the emphasis placed on relative energetics and reaction pathways at mineral edge sites. Electrolyte ions are included only to define solution composition and maintain charge neutrality, and we avoid drawing conclusions that would depend sensitively on the detailed description of ion dispersion interactions. Importantly, as demonstrated in Supplementary Section 2.4, the resulting MACE potential accurately reproduces AIMD free-energy profiles for proton transfer under both excess-proton and proton-deficient conditions, with excellent agreement across the entire reaction coordinate.[3,4] Since proton transfer and proton mobility are the central mechanisms governing charge buffering and pH effects at the clay–water interface, this validation directly supports the suitability of the chosen reference level for the phenomena investigated here, rather than the detailed description of spectator counterions.

We also note that previous studies have shown that, despite its known limitations, revPBE-D3 can yield internally consistent results for aqueous and interfacial systems when numerical convergence is carefully controlled.[12] In our calculations, a plane-wave cutoff of 1200 Ry, which is substantially higher than the minimum cutoff required to converge forces on Na reported in the literature, was employed to ensure robust force convergence within the chosen functional framework.

Finally, we emphasize that the goal of this study is to elucidate proton transfer and acid–base reactivity at montmorillonite edge sites, rather than to resolve detailed ion hydration structures or adsorption thermodynamics. We have clarified this scope and its associated limitations in the revised Supplementary Information.

" While revPBE-D3 has known limitations for isolated cation hydration, the present focus on edge reactivity at clay–water interfaces motivates its use as a consistent and computationally tractable reference for generating reactive training data, consistent with widely used clay force fields[13] and supported by our validation of clay structures, bulk water, and proton transfer free-energy profiles. "

R2. RESPONSE TO REFEREE 2

Referee's Summary

Recommendation: This paper is probably publishable, but major revision is needed; I do not need to see future revisions.

Comments: I have read the the article, ”Clay Edges Are Dynamic Proton-conducting Networks Modulated by Structure and pH” with great interest. This is a nice study with potentially interesting findings relevant to the geochemical importantly the colloidal community.

My first impression is that this too long and too comprehensive for a JPC-letter. This is a an issue for the editor to resolve. I have made a note to him.

Nevertheless it is a nice study, but no simple message that is required for letters (see guidelines) is conveyed. A letter style result would be to say something definitive regarding the pKa (under solution conditions) for these important systems regarding curvature, size, and even the loading of the defects. This would have profound implications for colloidal studies of different size nanoparticle and the plethora of phenomena related to charge-charge attractions and surface charge regulation as they stated early in the manuscript.

What is presented is a very high quality and detailed study of small (very small) nanoparticles under ”pH.” First, I think the authors should embrace a ”notional” scale rather than an absolute values they state. You can't determine the system pH without rigorous analyses of simulations in conjunction with non-ideal solution thermodynamics and addressing how the activity of H_3O^+ is related to the concentration of H_3O^+. In fact, if the authors state they can do this, this would be a topic worthy of a letter contribution. The setup appears to be composition-defined (fixed numbers of H_3O^+/Cl^- or OH^-/Na^+ added initially). Because the clay edge buffers protons, the effective acidity can drift during the trajectory, consistent with their tracking of net proton excess
(#H_3O^+ - #OH^-).

What I liked about the manuscript:

1. *The isomorphic substitution (Al/Mg) results are fascinating. This is a highlight of the study.One could write a short letter about this phenomena.*
2. *The finding of less proton hopping events in acidic conditions, although a very interesting andsolid finding, seems rather intuitive. Again, I think have a molecular verification of intuition via simulation is an important self-consistent check, it doesn't strike me as s a ”letter” quality result. 3. The resulting rich proton dynamics on edges vs surfaces is also very interesting.*

In summary the work is of high quality and I would accept this immediately with the suggested improvements if it were a standard JPC contribution. It's an important study by a fantastic group. My complaints are the JPC-letter format. It seems forced. This

figures are nice but huge and complex. It's a detailed read with lots of rich and important details. Again, my only complaint is that it is too much for a latter journal. If the other referees and the Editor agree this is a proper letter I will go along. It is an important and well executed study that the practitioners in the field will enjoy and cite.

Response to Summary

We thank the reviewer for the thoughtful and detailed evaluation and for recognizing the quality and potential impact of the study. We acknowledge the concern that the manuscript is currently too long and comprehensive, with figures and analyses that risked obscuring the central message. We agree that, in its original form, the Letter format felt forced.

In response, we have substantially restructured the manuscript to sharpen its focus and improve accessibility. The revised version is now organized around a single conceptual message: that montmorillonite edge surfaces form amphoteric, dynamically adaptive proton-exchange networks whose reactive state is modulated by the local chemical environment. To achieve this, we have consolidated the discussion of pH effects and isomorphic substitution into a single section on environmental modulation of proton transfer dynamics, and relocated detailed analyses of individual proton transfer pathways, free-energy profiles, and site-specific energetics to the Supplementary Information.

As a result, the main text now emphasizes the key physical picture and its broader implications for surface reactivity, rather than exhaustive mechanistic detail. We believe these changes directly address the reviewer's concern regarding scope and presentation, and that the revised manuscript now more clearly aligns with the expectations of a *The Journal of Physical Chemistry Letters* contribution while retaining the scientific depth of the work.

Comment 2.1.

As stated above, the manuscript would benefit from explicitly defining pH and from reporting how much this metric changes over time (and over the analysis window). But, there is value to saying that the study takes place on a "notional" scale of "low" , "medium" (near neutral) and "high" pH environments and would relabel their figures.

Response to Comment 2.1.

We fully agree with the reviewer's assessment. The simulations are composition-defined rather than rigorously pH-defined, and the effective acidity cannot be uniquely determined at all times without invoking non-ideal solution thermodynamics and activity coefficients. As noted by the reviewer, proton buffering by the clay edge surfaces leads to time-dependent changes in the aqueous proton population, which we explicitly track via the net proton excess.

Because the number of ions in the simulation cell is small, any attempt to compute an instantaneous pH would result in large, nonphysical fluctuations and could be misleading. For this reason, the quoted pH values are used only to describe the initial solution composition and to provide an intuitive reference. We agree that a notional description in terms of low, near-neutral, and high acidity environments is more appropriate.

To explicitly address this point in the manuscript, we have added the following clarifying text:

" To examine the pH-dependent reactivity of edge sites, each nanoparticle was simulated in aqueous environments prepared at initial acidic (pH 0.44), neutral (pH 7), and basic (pH 13.56) conditions (Figures 1d–f), including respective counter ions to retain charge neutrality. These pH values represent the equivalent bulk pH associated with the initial concentrations of hydronium or hydroxide ions in the simulation cell and are provided as a reference for comparison. During the simulations, no external buffering or ion exchange was imposed to maintain a fixed pH; instead, proton exchange between the aqueous phase and the clay edge surfaces was allowed to proceed freely, leading to time-dependent changes in the effective acidity of the solution. Accordingly, the terms acidic, neutral, and basic environments are used throughout to denote these composition-defined conditions rather than strictly controlled macroscopic pH values. "

Moreover, in the revised manuscript, we have now reduced emphasis on absolute pH values, clarified that acidity is defined by the initial composition, and relabeled figures and discussion to focus on acidic, neutral, and basic environments. This approach better reflects the physical meaning of the simulations and avoids overinterpretation of pH in a finite, reactive system.

Comment 2.2.

It should be feasible to analyze a representative subset of the existing trajectories to quantify surface charge-density changes for the studied systems. This would be of broad interest to the geochemistry community.

Response to Comment 2.2.

We thank the reviewer for this suggestion. Following this comment, we have explicitly analyzed the timedependent evolution of the nanoparticle surface charge density by accounting for both the intrinsic structural charge from isomorphic substitution and dynamic protonation/deprotonation at edge sites. The resulting surface charge evolution is now shown in Fig. R3b and discussed in detail in the revised manuscript. Together with the aqueous proton excess (Fig. R3a), this analysis provides a quantitative, molecular-level description of surface charge regulation and buffering behavior. The changes in the revised manuscript are as follows,

“ The evolution of the aqueous proton population and the surface charge of the montmorillonite nanoparticle represent two complementary views of the same proton-exchange process, as shown in Figs. R3a and b. Changes in the number of hydronium and hydroxide ions in solution are necessarily mirrored by protonation and deprotonation events at the edge surfaces, leading to a dynamic redistribution of charge between the aqueous phase and the nanoparticle. Under acidic conditions, proton uptake at edge functional groups increases the positive surface charge density, whereas under basic conditions, deprotonation of surface hydroxyls enhances the net negative surface charge. Together, these results demonstrate that the clay nanoparticle and the surrounding solution form a coupled reactive system in which reversible proton exchange gives rise to pH-dependent surface charge regulation and buffering behavior, reflecting the amphoteric character of the nanoparticle. ”

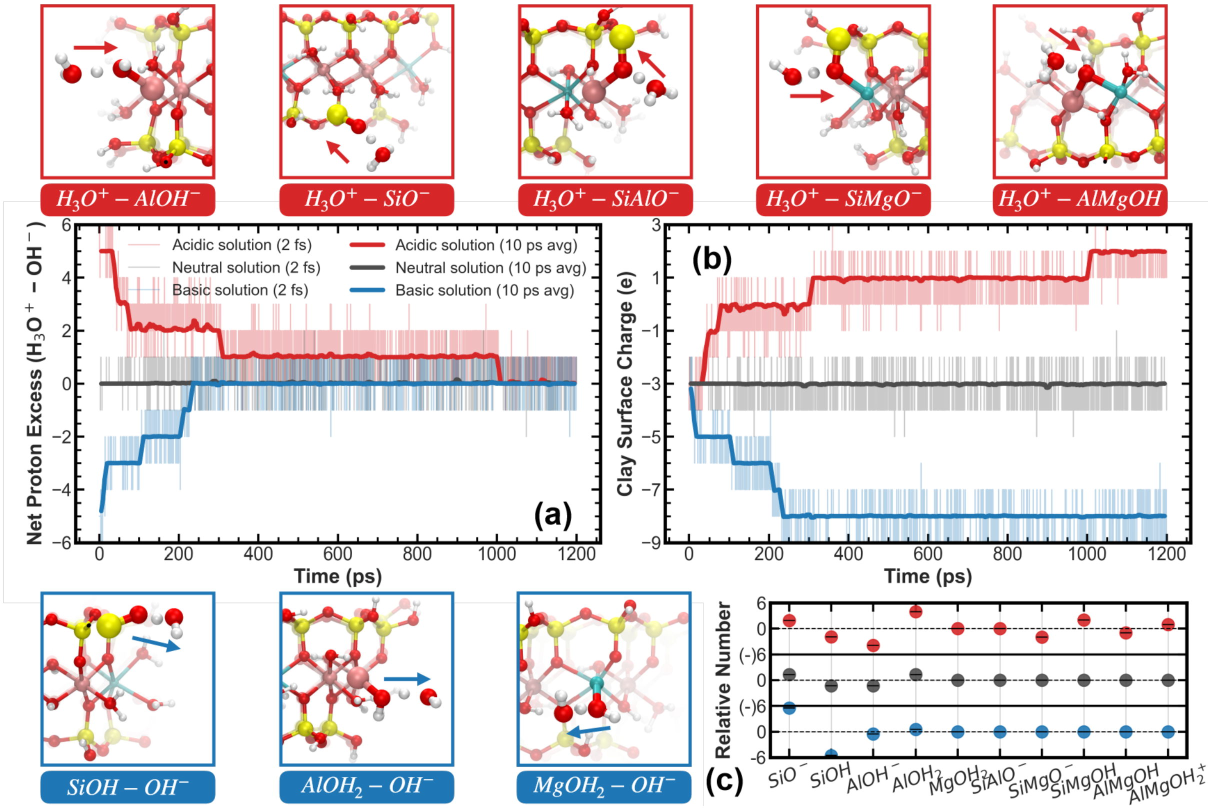

Figure R3: Protonation and deprotonation behavior of the montmorillonite nanoparticle in different aqueous environments. (a) Time evolution of the net proton excess in the aqueous phase (defined as the number of hydronium ions minus hydroxide ions) over 1200 ps. (b) Time evolution of the net surface charge of the montmorillonite nanoparticle, derived from the intrinsic negative structural charge of the clay due to isomorphic substitution, together with protonation and deprotonation of the nanoparticle through proton exchange with the aqueous solution. Transparent curves represent data sampled every 2 fs; solid lines denote a 10 ps average. (c) Relative abundance of various surface functional groups during the final 200 ps, compared to their initial populations; error bars are included but smaller than the symbol size. In all panels, colors denote the solution pH: red for acidic, black for neutral, and blue for basic conditions. Top panel: Surface groups that gain protons from hydronium ions under acidic conditions. Bottom panel: Surface groups that donate protons to hydroxide ions under basic conditions. Arrows indicate the direction of proton transfer, with representative surface reactions labeled below each group pair. Results shown correspond to one representative montmorillonite nanoparticle; analogous behavior for the other two configurations is provided in the Supplementary Information.

Comment 2.3.

How sensitive are the reported conclusions to the choice of ML potential architecture and training framework? In particular, do the free energies remain consistent when using an alternative ML engine/potential (trained to comparable reference data and accuracy) rather than MACE?

Response to Comment 2.3.

We thank the reviewer for this important methodological question. The sensitivity of the reported results to the choice of machine-learning potential (MLP) framework is a valid concern in reactive molecular simulations.

In the present work, we employ MACE as a state-of-the-art equivariant MLP framework that offers an excellent balance between accuracy, training efficiency, and long-time dynamical stability. More importantly, recent systematic benchmarks have demonstrated that, when trained to comparable first-principles reference data and accuracy, different modern MLP architectures yield highly consistent structural, thermodynamic, and dynamical properties. In particular, a recent comprehensive study comparing multiple leading MLP frameworks (including MACE, GRACE, SevenNet, MatterSim, and ORB) showed that training effectively harmonizes performance across architectures, leading to near *ab initio* accuracy and only minor framework-dependent differences in practice.[14]

To clarify this point, we have added a brief statement to the Methods section:

" Recent systematic benchmarks have demonstrated that, when trained to comparable first-principles reference data and accuracy, different modern machine-learning potential architectures yield highly consistent structural, thermodynamic, and dynamical properties, with only minor framework-dependent differences in practice.[14] "

Our conclusions are based primarily on relative free energies, proton transfer pathways, and pH-dependent trends, which are governed by the overall shape of the underlying potential energy surface rather than by subtle architecture-specific details. As long as the MLP faithfully reproduces the same first-principles reference data within comparable error bounds, these relative quantities are expected to be robust with respect to the specific ML engine used.

To further promote transparency and reproducibility, we plan to make the training datasets publicly available, enabling future comparisons with alternative MLP frameworks trained to the same reference data. We therefore expect the reported mechanistic insights and free-energy trends to be transferable beyond the specific choice of MACE.

We sincerely thank all reviewers for their constructive and insightful comments. We believe that the additions we made to the manuscript based on all reviewers' comments considerably improved the manuscript.

REFERENCES

[1]X. Liu, X. Lu, M. Sprik, J. Cheng, E. J. Meijer, and R. Wang, "Acidity of edge surface sites of montmorillonite and kaolinite," Geochimica et Cosmochimica Acta **117**, 180–190 (2013).

[2]X. Liu, J. Cheng, M. Sprik, X. Lu, and R. Wang, "Surface acidity of 2:1-type dioctahedral clay minerals from first principles molecular dynamics simulations," Geochimica et Cosmochimica Acta **140**, 410–417 (2014).

[3]L. B. Skinner, C. Huang, D. Schlesinger, L. G. M. Pettersson, A. Nilsson, and C. J. Benmore, "Benchmark oxygen-oxygen pair-distribution function of ambient water from x-ray diffraction measurements with a wide q-range," The Journal of Chemical Physics **138**, 074506 (2013).

[4]X. R. Advincula, K. D. Fong, A. Michaelides, and C. Schran, "Protons accumulate at the graphene–water interface," ACS Nano **19**, 17728–17737 (2025).

[5] H. J. Ploehn and C. Liu, “Quantitative analysis of montmorillonite platelet size by atomic force microscopy,” Industrial & Engineering Chemistry Research **45**, 7025–7034 (2006).
[6] S. Ali and R. Bandyopadhyay, “Effect of electrolytes on the microstructure and yielding of aqueous dispersions of colloidal clay,” Soft Matter **12**, 414–421 (2016).
[7] T. R. Underwood and I. C. Bourg, “Large-scale molecular dynamics simulation of the dehydration of a suspension of smectite clay nanoparticles,” The Journal of Physical Chemistry C **124**, 3702–3714 (2020).
[8] D. M. S. Martins, M. Molinari, M. A. Gonc¸alves, J. P. Mir˜ao, and S. C. Parker, “Toward modeling clay mineral nanoparticles: The edge surfaces of pyrophyllite and their interaction with water,” The Journal of Physical Chemistry C **118**, 27308–27317 (2014).
[9] J. Li, H. Ma, Y. Yan, J. Zhang, and Z. Li, “Molecular insights into the aggregation mechanism of montmorillonite colloid due to calcium contamination: A molecular dynamics simulation study,” Applied Clay Science **247**, 107191 (2024).
[10] J. L. Suter, L. Kabalan, M. Khader, and P. V. Coveney, “Ab initio molecular dynamics study of the interlayer and micropore structure of aqueous montmorillonite clays,” Geochimica et Cosmochimica Acta **169**, 17–29 (2015).
[11] S. V. Churakov, “Structure and dynamics of the water films confined between edges of pyrophyllite: A first principle study,” Geochimica et Cosmochimica Acta **71**, 1130–1144 (2007).
[12] N. O’Neill, B. X. Shi, K. Fong, A. Michaelides, and C. Schran, “To pair or not to pair? machine-learned explicitly-correlated electronic structure for nacl in water,” The Journal of Physical Chemistry Letters **15**, 6081–6091 (2024).
[13] R. T. Cygan, J. J. Liang, and A. G. Kalinichev, “Molecular models of hydroxide, oxyhydroxide, and clay phases and the development of a general force field,” Journal of Physical Chemistry B **108**, 1255–1266 (2004).
[14] M. Radova, W. G. Stark, C. S. Allen, R. J. Maurer, and A. P. Bart´ok, “Fine-tuning foundation models of materials interatomic potentials with frozen transfer learning,” npj Computational Materials **11**, 237 (2025).

jz-2025-03748z.R2

Name: Peer Review Information for "Clay Edges Are Dynamic Proton-conducting Networks Modulated by Structure and pH"

Second Round of Reviewer Comments

Reviewer: 1

Comments to the Author

The authors have mostly addressed my concerns.

However, they have not computed what they call the "static pKa" of OH group sat mineral surfaces using their MLFF. This is what AIMD pKa calculations do. Therefore they cannot claim that their results "move beyond previous static or short-timescale simulations by capturing ..." (right column, first full paragraph, page 9). Their results are merely

complimentary to existing existing AIMD pKa calculations, perhaps consistent with them up to +/- 1 pH unit range.

Somewhere in the manuscript they *must* also state explicitly that they have not compared "static pKa" values with AIMD simulations under the same conditions. Frankly, the absence of such comparisons makes me very uncomfortable with the authors' MLFF, and about recommending this paper for publication. But if they state explicitly that they have *not* made this comparison, the readers can at least judge this for themselves.

If the authors make these two changes, I recommend publication in the Journal of Physical Chemistry Letters.

Author's Response to Peer Review Comments:

(Dated: 9 February 2026)

Response to Reviewers' Comments

The reviewer reports are repeated in full length *in italics*, respond in a point-by-point manner to all questions and suggestions, and show in red added or changed text in the manuscript.

RESPONSE TO THE COMMENTS OF REVIEWER #1

The authors have mostly addressed my concerns.

However, they have not computed what they call the "static pKa" of OH group sat mineral surfaces using their MLFF. This is what AIMD pKa calculations do. Therefore they cannot claim that their results "move beyond previous static or short-timescale simulations by capturing ..." (right column, first full paragraph, page 9). Their results are merely complimentary to existing existing AIMD pKa calculations, perhaps consistent with them up to +/- 1 pH unit range.

*Somewhere in the manuscript they *must* also state explicitly that they have not compared "static pKa" values with AIMD simulations under the same conditions. Frankly, the absence of such comparisons makes me very uncomfortable with the*

*authors' MLFF, and about recommending this paper for publication. But if they state explicitly that they have *not* made this comparison, the readers can at least judge this for themselves.*

If the authors make these two changes, I recommend publication in the Journal of Physical Chemistry Letters.

We thank the reviewer for this important and insightful comment. While suggesting in the comment to address this point by clarifying the scope of our claims, we have taken this feedback as an opportunity to go further to validate our MLFF and broaden the message of the manuscript. In direct response to this point, we have now performed additional enhanced-sampling simulations to explicitly determine the pK_a values of representative surface groups that have been widely explored in the AIMD literature[1,2]. These new calculations enable a direct and quantitative comparison with existing AIMD-based pK_a results under comparable conditions. We note that these new calculations constitute a substantial additional simulation effort: for each surface group, pK_a values were obtained from umbrella sampling using ten biasing windows, and three distinct surface terminations were considered. In addition, the reference water self-dissociation reaction was computed using 30 umbrella windows at 300 K and repeated at four temperatures (300, 330, 360, and 390 K), yielding a total of over 100 enhanced-sampling simulations. Overall, these new simulations show that the MLFFderived pK_a values agree with established AIMD benchmarks under comparable conditions and demonstrate that our long-timescale enhanced-sampling approach can deliver robust, quantitative pK_a estimates at mineral–water interfaces, thereby substantially strengthening the manuscript. The methodology, results, and analysis of these new pK_a calculations are presented in a new Section S2.6 of the Supplementary Information.

Validation of Montmorillonite Edge Sites pK_a

To further assess whether the machine learning potential developed in this work can faithfully describe interfacial acid–base chemistry, we compute pK_a values for representative edge surface groups of montmorillonite and compare them directly with values reported in previous AIMD studies[1]. To this end, pK_a values are obtained from free-energy profiles describing the deprotonation of different surface groups, constructed using umbrella sampling. To limit the structural heterogeneity associated with the full nanoparticle model and to enable direct comparison with previous AIMD calculations, we adopt a simplified slab representation exposing the (010) edge with the relevant surface terminations, as shown in Figure R1.

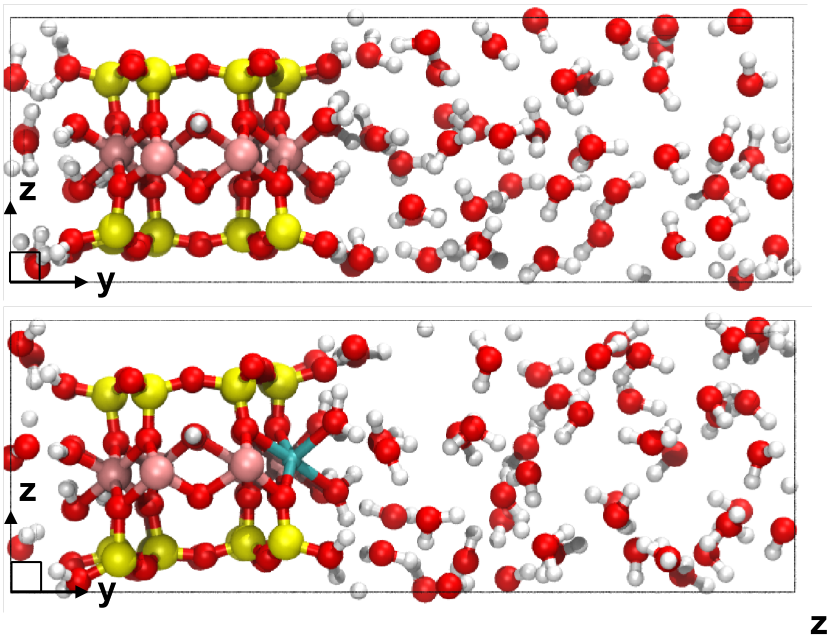

Fig. R1. Slab models used for the pK_a calculations. The top panel shows the system without isomorphic substitution, while the bottom panel includes Mg-for-Al isomorphic substitution in the octahedral sheet. Atom colors are as follows: O (red), H (white), Si (yellow), Al (pink), and Mg (cyan).

The deprotonation free-energy profiles are constructed by biasing a collective variable that quantifies proton coordination to a selected surface oxygen atom, O^*. Specifically, within each umbrella window, sampling is performed by varying the coordination number of O^* with respect to all hydrogen atoms in the simulation cell, as defined below:

$$n_{\mathrm{O^*-H}} = \sum_{i=1}^{N} \frac{1-(r_i/R_0)^{12}}{1-(r_i/R_0)^{24}} \tag{1}$$

where the sum runs over all hydrogen atoms in the simulation cell, r_i is the distance between hydrogen i and O^*, and R_0 is a characteristic switching distance fixed at 1.38 ˚A. Sampling along this reaction coordinate is enforced by restraining $n_{\mathrm{O^*-H}}$ around a target value n'_{H} using a harmonic bias with a force constant of 500 kcal mol^{-1} per squared coordination unit. The resulting free-energy profiles are reconstructed by umbrella integration[3], and the pK_a values are obtained using the following expression,

$$pK_a = \frac{\Delta F_{site}}{RT \cdot \ln(10)} + \left(14 - \frac{\Delta F_{\mathrm{H_2O}}}{RT \cdot \ln(10)}\right) \tag{2}$$

where ΔF_{site} is the free-energy difference between the protonated and deprotonated states of the surface site, R is the ideal gas constant, T is the temperature (here, 300 K), and $\Delta F_{\mathrm{H_2O}}$ denotes the free energy associated with water self-dissociation.

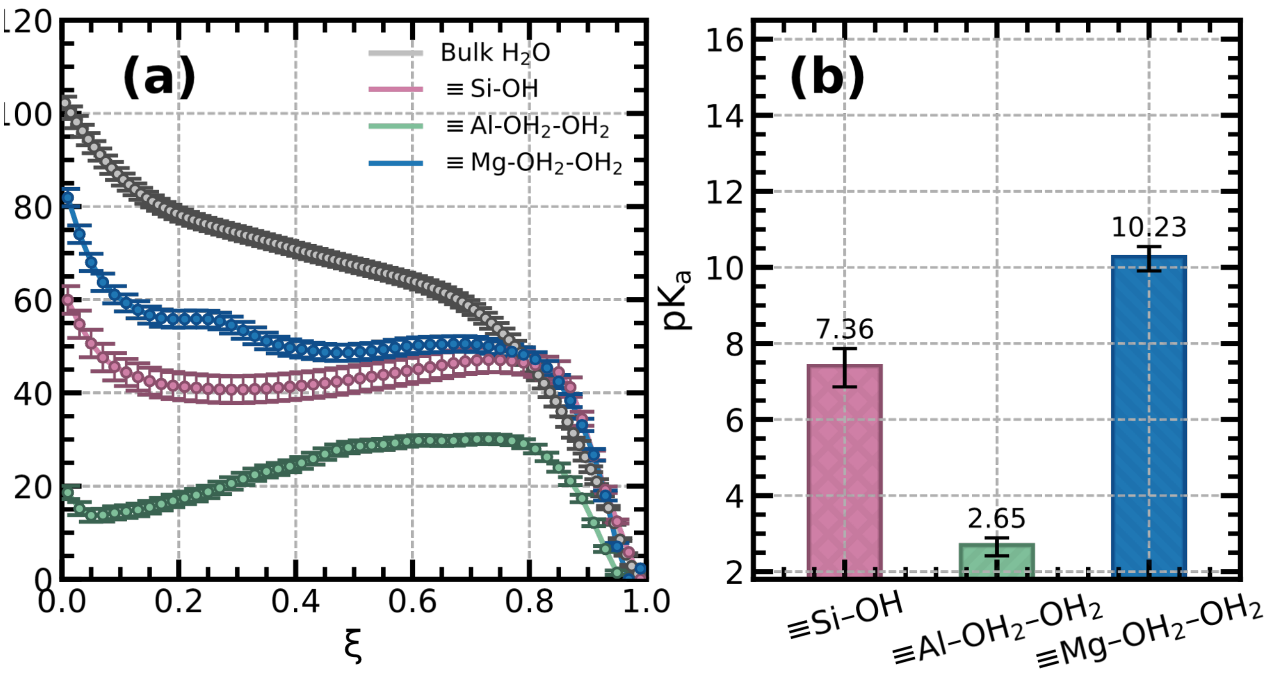

Fig. R2. (a) Free-energy profiles for the deprotonation of the ≡Si–OH, ≡Al–OH_2–OH_2, and ≡Mg–OH_2–OH_2 surface groups, together with the reference water self-dissociation reaction. The reaction coordinate ξ corresponds to the coordination number n_{O*-H} for the ≡Si–OH group, and to $n_{O*-H}-1$ for the ≡Al–OH_2–OH_2, ≡Mg–OH_2–OH_2, and bulk H_2O reaction, allowing all profiles to be displayed on a common axis. (b) pK_a values obtained from the free-energy differences using Eq. 2. All free-energy profiles were obtained from umbrella-sampling simulations using umbrella integration. For each surface group, ten umbrella windows were employed, with simulation lengths of 250 ps per window. The reference water self-dissociation reaction was computed using 30 umbrella windows at 300 K.

TABLE I. pK_a values of edge surface groups of montmorillonite.

	≡Si–OH	≡Al–OH_2OH_2	≡Mg–OH_2OH_2
pK_a (Ref. 1)	7.0 ± 0.7	3.1 ± 0.5	13.2 ± 0.5
pK_a (MLP)	7.4 ± 0.5	2.7 ± 0.3	10.2 ± 0.3

The deprotonation free-energy profiles obtained for the ≡Si–OH, ≡Al–OH_2–OH_2, and ≡Mg–OH_2–OH_2 surface groups are presented in Figure R2, together with a quantitative comparison to available AIMD results in Table I. Across all surface terminations, we observe close agreement with previous AIMD estimates, supporting the ability of the machine learning potential developed in this work to reliably describe acid–base reactions at mineral–water interfaces. While these pK_a values agree well with AIMD benchmarks, they are derived from finite-system free-energy differences along a chosen collective variable and referenced to water self-dissociation, so they remain sensitive to the reaction coordinate definition and any residual standard-state corrections implicit in the slab geometry. We estimate that these methodological choices introduce an additional uncertainty of order 1 pH unit, comparable to typical variations between different pK_a protocols and the underlying electronic-structure

theory for mineral–water interfaces. In particular, whereas earlier AIMD studies primarily employed the vertical energy gap method[1], the present work relies on umbrella sampling to avoid the use of dummy particles important to be compatible with the current MLP framework.

When computing the pK_a values of the ≡Si–OH, ≡Al–OH_2–OH_2, and ≡Mg–OH_2–OH_2 surface groups, it is important to note that all values are referenced to the deprotonation free energy of hydronium, corresponding to the water self-dissociation reaction, following established practice in previous studies[1,4,5]. The free-energy profile for water self-dissociation is shown in grey in Figure R2 and is itself in excellent agreement with earlier AIMD results[6,7]. Using the relation $pK_w = \Delta F^{\ddagger}/(RT \cdot \ln 10)$, where $\Delta F^{\ddagger}$ is the free energy barrier between reactant and product states, we obtain a pK_w of 13.9 ± 0.1 at 300 K. As a further validation of the MLP, we also examined its ability to reproduce the temperature dependence of water self-dissociation. As shown in Figure R3, free-energy barriers obtained using the MACE potential closely follow the corresponding AIMD results across the full temperature range considered[6]. Moreover, the enthalpic and entropic contributions extracted from the temperature dependence of the barriers are in excellent agreement between the two approaches, indicating that the MLP captures not only accurate free-energy barriers but also the underlying thermodynamic balance governing the self-dissociation process.

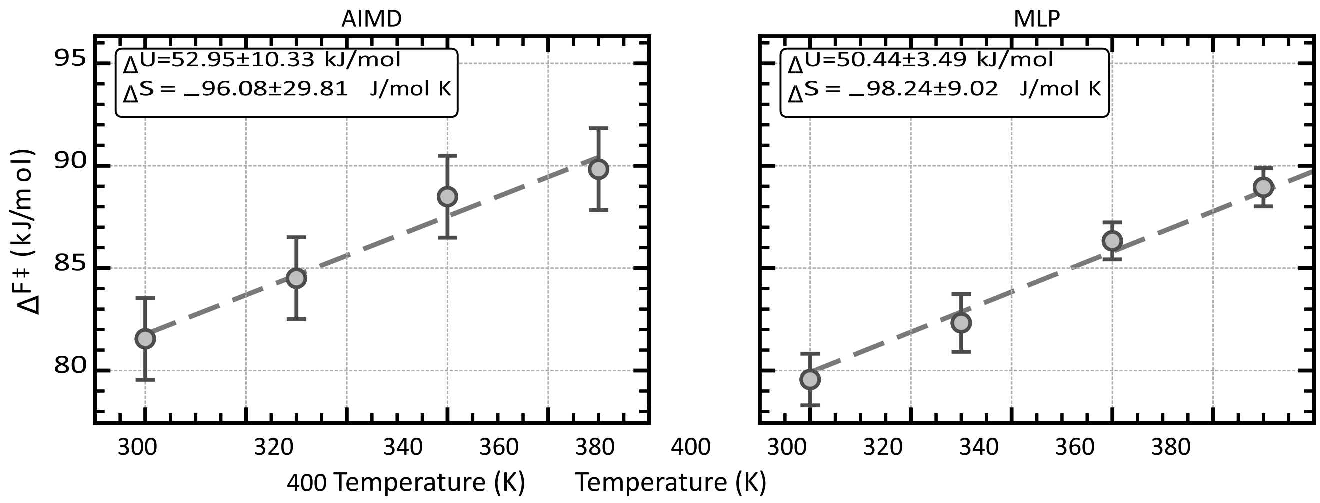

Fig. R3. Comparison of temperature-dependent free energy barriers for water self-dissociation computed using AIMD[6] and the MLP developed in this work. The free energy barrier between reactant and product states, $\Delta F^{\ddagger}$, is shown as a function of temperature T for AIMD (left) and MACE (right), and fitted using the relation $\Delta F = \Delta U - T\Delta S$ to extract the enthalpic and entropic contributions. The close agreement between the two methods highlights the accuracy of the MLP in describing the dissociation process.

Additionally, we have added the following statement in the main text to reflect these changes.

" Further validation confirmed the model's reliability in reproducing interfacial energetics, clay lattice structures, bulk water properties, and proton transfer free energies, including pK_a values for representative surface terminations consistent with previous AIMD studies[1] and

an accurate description of water self-dissociation in line with literature estimates[6,7] (see Supplementary Section S2)."

With these additions, we have also revised the wording in the right column of page 9 to more accurately reflect the scope and positioning of the work.

" Our results complement previous static and short-timescale simulations by explicitly capturing the dynamic and recurrent nature of surface proton-transfer reactions, including inter-site mechanisms that are difficult to access with static approaches."

The revised manuscript now clearly distinguishes between the computation of pK_a values, which can be directly compared with AIMD-based pK_a estimates, and the additional dynamical insight enabled by the substantially longer timescales accessible with the MLFF.

We hope that the explicit inclusion of the pK_a calculations and the revised framing of the results address the reviewer's concerns regarding the reliability of the MLFF and provide a clearer basis for assessing its performance. We believe that these additions substantially strengthen the manuscript and would like to thank the referee for pushing us to add these important benchmarks.

[1]X. Liu, X. Lu, M. Sprik, J. Cheng, E. J. Meijer, and R. Wang, Geochimica et Cosmochimica Acta **117**, 180 (2013).

[2]X. Liu, X. Lu, E. J. Meijer, R. Wang, and H. Zhou, Geochimica et Cosmochimica Acta **81**, 56 (2012).

[3]J. K¨astner and W. Thiel, The Journal of Chemical Physics **123**, 144104 (2005).

[4]I. Ivanov, B. Chen, S. Raugei, and M. L. Klein, The Journal of Physical Chemistry B **110**, 6365 (2006).

[5]R. Wang, V. Carnevale, M. L. Klein, and E. Borguet, The Journal of Physical Chemistry Letters **11**, 54 (2020).

[6]Y. Litman and A. Michaelides, Journal of the American Chemical Society **147**, 44885 (2025).

[7]X. R. Advincula, Y. Litman, K. D. Fong, W. C. Witt, C. Schran, and A. Michaelides, (2025), arXiv:2508.13034 [physics.chem-ph].
